# DNA priming and gp120 boosting induces HIV-specific antibodies in a randomized clinical trial

**DOI:** 10.1172/JCI128699

**Published:** 2019-09-30

**Authors:** Nadine G. Rouphael, Cecilia Morgan, Shuying S. Li, Ryan Jensen, Brittany Sanchez, Shelly Karuna, Edith Swann, Magdalena E. Sobieszczyk, Ian Frank, Gregory J. Wilson, Hong-Van Tieu, Janine Maenza, Aliza Norwood, James Kobie, Faruk Sinangil, Giuseppe Pantaleo, Song Ding, M. Juliana McElrath, Stephen C. De Rosa, David C. Montefiori, Guido Ferrari, Georgia D. Tomaras, Michael C. Keefer, the HVTN 105 Protocol Team and the NIAID HIV Vaccine Trials Network

**Affiliations:** 1 Hope Clinic of the Emory Vaccine Center, Division of Infectious Diseases, Emory University, Atlanta, Georgia, USA.; 2 Vaccine and Infectious Disease Division, Fred Hutchinson Cancer Research Center, Seattle, Washington, USA.; 3 Division of AIDS, NIH, Bethesda, Maryland, USA.; 4 Columbia University Medical Center, New York, New York, USA.; 5 University of Pennsylvania, Philadelphia, Pennsylvania, USA.; 6 Vanderbilt University Medical Center, Nashville, Tennessee, USA.; 7 New York Blood Center, New York, New York, USA.; 8 University of Washington, Seattle, Washington, USA.; 9 UCSF, San Francisco, California, USA.; 10 Department of Medicine, University of Rochester School of Medicine & Dentistry, Rochester, New York, USA.; 11 Global Solutions for Infectious Diseases, South San Francisco, California, USA.; 12 Division of Immunology and Allergy, Lausanne University Hospital (CHUV), Lausanne, Switzerland.; 13 EuroVacc Foundation, Lausanne, Switzerland.; 14 Department of Surgery, Duke University Medical Center, Durham, North Carolina, USA.; 15 The HVTN 105 Protocol Team and the NIAID HIV Vaccine Trials Network is detailed in the Supplemental Acknowledgments.

**Keywords:** AIDS/HIV, Vaccines, AIDS vaccine

## Abstract

**BACKGROUND:**

RV144 is the only preventive HIV vaccine regimen demonstrating efficacy in humans. Attempting to build upon RV144 immune responses, we conducted a phase 1, multicenter, randomized, double-blind trial to assess the safety and immunogenicity of regimens substituting the DNA-HIV-PT123 (DNA) vaccine for ALVAC-HIV in different sequences or combinations with AIDSVAX B/E (protein).

**METHODS:**

One hundred and four HIV-uninfected participants were randomized to 4 treatment groups (T1, T2, T3, and T4) and received intramuscular injections at 0, 1, 3, and 6 months (M): T1 received protein at M0 and M1 and DNA at M3 and M6; T2 received DNA at M0 and M1 and protein at M3 and M6; T3 received DNA at M0, M1, M3, and M6 with protein coadministered at M3 and M6; and T4 received protein and DNA coadministered at each vaccination visit.

**RESULTS:**

All regimens were well tolerated. Antibodies binding to gp120 and V1V2 scaffold were observed in 95%–100% of participants in T3 and T4, two weeks after final vaccination at high magnitude. While IgG3 responses were highest in T3, a lower IgA/IgG ratio was observed in T4. Binding antibodies persisted at 12 months in 35%–100% of participants. Antibody-dependent cell-mediated cytotoxicity and tier 1 neutralizing-antibody responses had higher response rates for T3 and T4, respectively. CD4^+^ T cell responses were detectable in all treatment groups (32%–64%) without appreciable CD8^+^ T cell responses.

**CONCLUSION:**

The DNA/protein combination regimens induced high-magnitude and long-lasting HIV V1V2–binding antibody responses, and early coadministration of the 2 vaccines led to a more rapid induction of these potentially protective responses.

**TRIAL REGISTRATION:**

ClinicalTrials.gov NCT02207920.

**FUNDING:**

National Institute of Allergy and Infectious Diseases (NIAID) grants UM1 AI068614, UM1 AI068635, UM1 AI068618, UM1 AI069511, UM1 AI069470, UM1 AI069534, P30 AI450008, UM1 AI069439, UM1 AI069481, and UM1 AI069496; the National Center for Advancing Translational Sciences, NIH (grant UL1TR001873); and the Bill & Melinda Gates Foundation (grant OPP52845).

## Introduction

Almost 40 million people are living with HIV worldwide. Every year, there are 1.8 million new infections and 1.2 million deaths from HIV-related causes (1), making the need for a safe and effective HIV vaccine a public health priority. To date, RV144 is the only vaccine efficacy trial that demonstrated protection against HIV acquisition (2). RV144 was a randomized placebo-controlled trial evaluating a regimen consisting of a replication-defective canarypox vector (ALVAC vCP1521) with HIV *gag*, *protease*, and *env* inserts administered at 0, 1, 3, and 6 months, in combination with a recombinant gp120 protein (AIDSVAX B/E) administered at 3 and 6 months among 16,402 adults at varying risk of HIV-1 acquisition in Thailand. Prior efficacy trials using AIDSVAX B/E (VAX003, ref. 3) or AIDSVAX B/B alone (VAX004, ref. 4) failed to protect against HIV acquisition, emphasizing the potential importance of a prime-boost regimen.

Vaccine efficacy in RV144, albeit moderate, appeared to be predominantly mediated by antibody responses, based on the results of a case-control study that examined immunogenicity in peripheral blood at the time of peak responses, 2 weeks after the final vaccination at month 6 (5). While vaccine efficacy was 31% at 36 months, the predetermined time point of primary analysis, early vaccine efficacy at 1 year was 60%, reflecting the kinetics of HIV-specific humoral responses in the peripheral blood (6). IgG antibodies directed against the variable loop region (V1V2) of gp120, including those of the IgG3 subclass, were associated with a lower risk of HIV-1 acquisition (5, 7). Specifically, the magnitude of anti-V1V2 IgG responses appeared to be important, as participants with titers in the highest tertile were found to have a vaccine efficacy of 60% compared with those in the lowest tertile or with negative responses (5, 6). In addition, HIV-specific antibody-dependent cell-mediated cytotoxicity (ADCC) in the presence of low HIV-1 envelope–specific (Env-specific) IgA correlated with decreased HIV-1 risk (5). Plasma HIV-1 Env–specific IgA antibodies in vaccine recipients were found to be directly correlated with infection risk in RV144 and were postulated to interfere with protective Fc-mediated antibody functions such as ADCC (5, 8). Thus, while these potential immunologic correlates of HIV infection risk require confirmation in ongoing efficacy trials (i.e., the HVTN 702/Uhambo study currently underway in South Africa), these immunologic markers can be helpful in early-phase studies to assess alternative vaccine strategies to optimize protective responses.

The HIV Vaccine Trials Network (HVTN) 105 trial (ClinicalTrials.gov NCT02207920) was designed to investigate vaccine priming alternatives to the RV144 strategy with a DNA-HIV candidate vaccine replacing the ALVAC vector and additional modifications of the prime-boost sequence. HVTN 105 evaluated the safety and immunogenicity of AIDSVAX B/E (consisting of a 1:1 mixture of gp120 strains MN and A244 in alum) also used in RV1144, and the DNA plasmid vaccine (DNA-HIV-PT123) containing a clade C (strain ZM96) gp140 envelope that was substituted for the ALVAC vCP1521 but contained the same HIV genes as ALVAC used in RV144. One HVTN 105 group (T3) matched the RV144 prime-boost regimen (DNA-HIV-PT123 at 0, 1, 3, and 6 months with AIDSVAX B/E at 3 and 6 months), while coadministration of both candidate vaccines at all 4 vaccination time points was examined in another group (T4). Another group (T2) matched the RV144 regimen but omitted the third and fourth DNA-HIV injections at 3 and 6 months, and the reverse of that sequence, protein vaccine given as a prime followed by DNA vaccine boosts, was examined in the remaining group (T1).

The DNA component of this experimental vaccine regimen offers several potential advantages over the ALVAC vector in RV144, in that DNA vaccines are thermostable, are relatively straightforward to manufacture, and provide more flexibility for vaccine design through formulation of multiple plasmids containing different HIV components and/or adjuvants in a single injection. DNA HIV vaccines also have a favorable safety profile in large studies (6) in combination with other vector vaccines such as modified vaccinia Ankara (MVA) (9) and adenovirus serotype 5 (10). To date, however, only 2 small HIV vaccine clinical trials have been conducted using combination regimens of DNA and recombinant protein, and these trials showed induction of significant and persistent binding antibodies and T cell responses (11, 12). Furthermore, to the best of our knowledge no human data exist on priming with a protein and boosting with a DNA vaccine and there are limited human data on the coadministration of these products from the initial vaccination, although studies in nonhuman primates show encouraging results (13–17).

The primary goal of the HVTN 105 trial was to utilize the immunologic profile of potential correlates of protection identified in the RV144 efficacy trial to determine (a) whether administration of a DNA-HIV vaccine instead of ALVAC-HIV in the same regimen can induce comparable or superior immune responses, and (b) how the early coadministration of DNA and protein affects the kinetics and character of immune responses over the course of the trial. In addition, HVTN 105 examined how 4 doses versus 2 doses of DNA-HIV priming affects immunogenicity, and whether protein immunization can serve as an effective prime for subsequent boosting with DNA-HIV.

## Results

### Study participants, demographics, and vaccine safety

**Figure 1:**
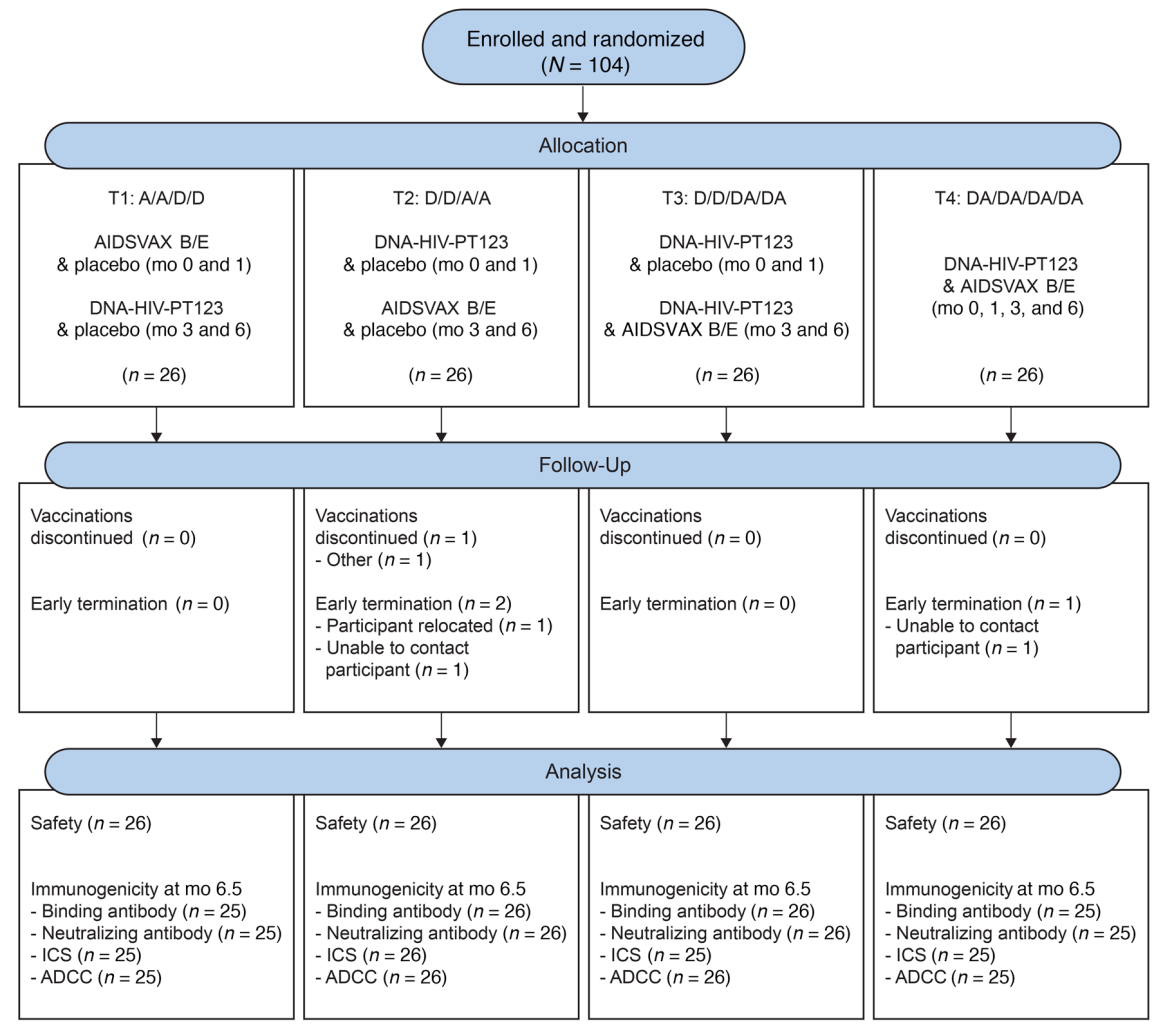
HVTN 105 CONSORT statement flow diagram.

One hundred and four participants at low risk for HIV acquisition were enrolled at US sites between July 16, 2014 and December 10, 2014, and randomly assigned into 1 of 4 study treatment groups (Table 1). Participant characteristics are described in Supplemental Table 1; supplemental material available online with this article; https://doi.org/10.1172/JCI128699DS1 Fifty-three percent of participants were male, 31% were non-white, and 10% were of Latino ethnicity. The median age of participants was 27 years (range 18–50 years). Demographics among the 4 groups were comparable. Ninety-eight percent of participants (102 of 104) received all 4 vaccinations and the overall retention rate in the trial was high (101 of 104, 97%); 1 participant (in T2) relocated and 2 participants (1 each in T2 and T4) were unable to be contacted during the follow-up period after receiving all 4 vaccinations (Figure 1).

Both DNA-HIV and AIDSVAX B/E vaccines were well tolerated. There were no serious adverse events (SAEs) reported in the study. Eighty-five percent (*n* = 88) of study participants experienced one or more adverse events (AEs), but only 9 (8.7%) of these participants had 15 AEs that were judged by the investigators to be related to the study agents (decrease in absolute neutrophil count [*n* = 3], elevation in alanine aminotransferase level [*n* = 3], decrease in hemoglobin level [*n* = 1], tenderness/enlargement of draining axillary lymph node [*n* = 3], pruritus at injection site [*n* = 2], nodule at injection site [*n* = 1], shoulder pain [*n* = 1], and fatigue [*n* = 3]). Local (Supplemental Figure 1A) and systemic (Supplemental Figure 1B) reactions, when present, were self-limited, with the majority being of mild to moderate grade. One participant in T4 experienced severe (grade 3) erythema of greater than 9 cm in diameter at the right deltoid (AIDSVAX B/E) injection site after the second and third vaccinations, and another participant in T2 reported grade 3 fatigue/malaise after the fourth vaccination. The most frequent local reactions were pain and/or tenderness in 85.6% (mild in 71.2%, moderate in 14.4%) of individuals, and the most frequent systemic reactions were malaise/fatigue in 40.4% (mild in 27.9%, moderate in 11.5%, severe 1%) of individuals. There were no statistically significant differences across the 4 groups for local or systemic reactions except more mild/moderate headache (*P* = 0.03) in T1, and no vaccinations were discontinued due to AEs.

### Vaccine-induced humoral immunogenicity

#### Frequency and magnitude of IgG, IgG subclass, and IgA Env-specific binding antibodies.

The total Env-specific binding-antibody responses were evaluated against vaccine strain gp120 antigens (ZM96.C, MN.B, and A244.AE) and consensus envelope antigens (Con S gp140 CFI [group M consensus]) central to all circulating strains, with good sensitivity for HIV vaccine immunogenicity assessment (7, 18, 19). We also evaluated IgG binding-antibody responses to different conformational V1V2 antigens that correlated with decreased risk of HIV acquisition in RV144, including those binding to C.1086 V1V2, the *env* sequence strain selected for further clinical trials in sub-Saharan Africa.

**Figure 2:**
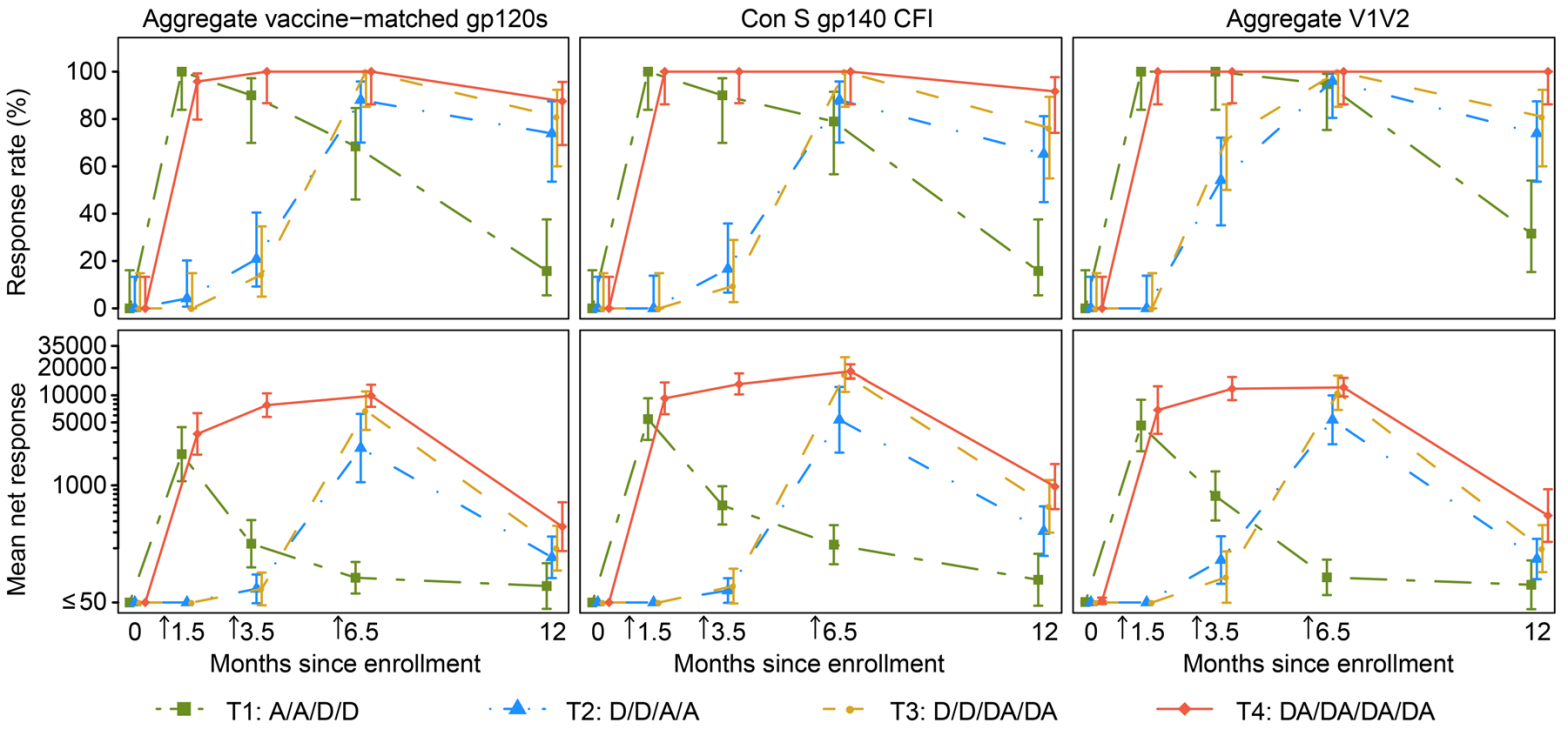
IgG binding-antibody responses in HVTN 105 participants over time, as measured by binding-antibody multiplex assay (BAMA) against aggregate vaccine-matched gp120 antigens, 1 gp140 antigen, and aggregate V1V2 antigens. Shown are the positive-response rates and 95% CIs estimated using the score test method (top panels) and the geometric mean response magnitudes among all participants and 95% CI based on an assumption of log(IgG) following a normal distribution (bottom panels) by time point and treatment group (*n* = 25, 26, 26, 25 in T1–T4, respectively). The lines connect the response rates/geometric mean magnitudes between time points. Vaccine-matched gp120 antigens: A244.AE, MN.B, and ZM96.C. gp140 antigen: Con S gp140 CFI. V1V2 antigens: 1086.C V1V2, CaseA2_gp70_V1V2.B, CaseA2_V1/V2/169K.B, and A244.AE V1V2. Arrows indicate the second, third, and fourth vaccinations. D, DNA; A, AIDSVAX B/E.

**Figure 3:**
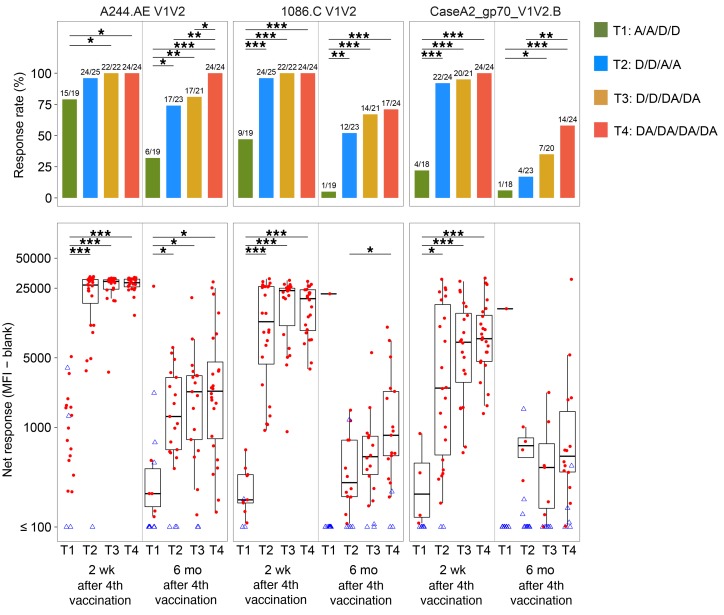
IgG binding-antibody responses 2 weeks and 6 months after the fourth vaccination in HVTN 105, as measured by binding-antibody multiplex assay (BAMA) against 3 V1V2 antigens. Shown are the positive-response rates (top panels) and the distribution of the response magnitudes (positive responders in red circles and nonresponders in blue triangles) and the box-and-whisker plots among the positive responders (the midline of the box-and-whisker plot denotes the median and the ends of the box-and-whisker plot denote the 25th and 75th percentiles) (bottom panels) by time point and treatment group (*n* = 25, 26, 26, 25 in T1-T4, respectively). V1V2 antigens: A244.AE V1V2, 1086.C V1V2, and CaseA2_gp70_V1V2.B. Bars on the top of plots indicate the significant differences between treatment groups within the same visit (*P ≤ 0.05; **P ≤ 0.01; ***P ≤ 0.001) without multiple-comparisons adjustment. The comparisons between treatment groups were performed using Fisher’s exact test for response rates and Wilcoxon’s rank-sum test for magnitudes. Fractions above bars on top panels indicate numbers of positive responses over total numbers of responses (negative and positive) by time point and treatment group. D, DNA; A, AIDSVAX B/E.

Within each treatment group, trends of binding-IgG response rates and geometric mean response magnitudes were similar over time across HIV antigens (vaccine-matched vs. consensus HIV envelopes, V1V2 antigens). In all treatment groups, response rates of 81%–100% were observed 2 weeks after the second vaccination with AIDSVAX B/E, which occurred at month 1.5 in T1 and T4 following early administration of protein, as opposed to month 6.5 in T2 and T3 (Figure 2). However, the response rates in T1 were not sustained at later time points with boosting by DNA alone. Comparing the other treatment groups at month 6.5 (2 weeks after the fourth vaccination), there were no significant differences between T3 and T4 in IgG response rates to any antigen, but significant differences were observed when T3 and T4 were compared to T2 for vaccine-matched antigens. T4 response rates were higher than T2 for ZM96.C (100% vs. 80%, *P* = 0.05) and A244.AE (100% vs. 80%, *P* = 0.05) (Supplemental Figure 2). Also, the IgG response rate was significantly higher in T3 than T2 for ZM96.C (100% vs. 80%, *P* = 0.05). Importantly, at this time point, binding-IgG responses to the HIV V1V2 antigens identified in RV144 as potential inverse correlates of risk (A244.AE V1V2 and 1086.C V1V2) were observed in 96% or more vaccinees in groups T2, T3, and T4, with high median response magnitudes (26,881–29,212 for A244.AE V1V2 and 11,723–23,634 for 1086.C V1V2) (Figure 3).

Durable binding-IgG responses, as measured at month 12 (6 months after the fourth vaccination), were demonstrated in over 65% of participants against the aggregated vaccine-matched and consensus envelope antigens only if they received a boosting regimen that included AIDSVAX B/E (groups T2, T3, and T4) (Figure 2). Although the magnitude of IgG responses against HIV V1V2 significantly decreased by month 12 (6 months after the fourth vaccination) in all groups, they were still detectable in the majority of T3 and T4 participants for A244.AE V1V2 (81% T3 and 100% T4) and 1086.C V1V2 (67% T3 and 71% T4).

**Figure 4:**
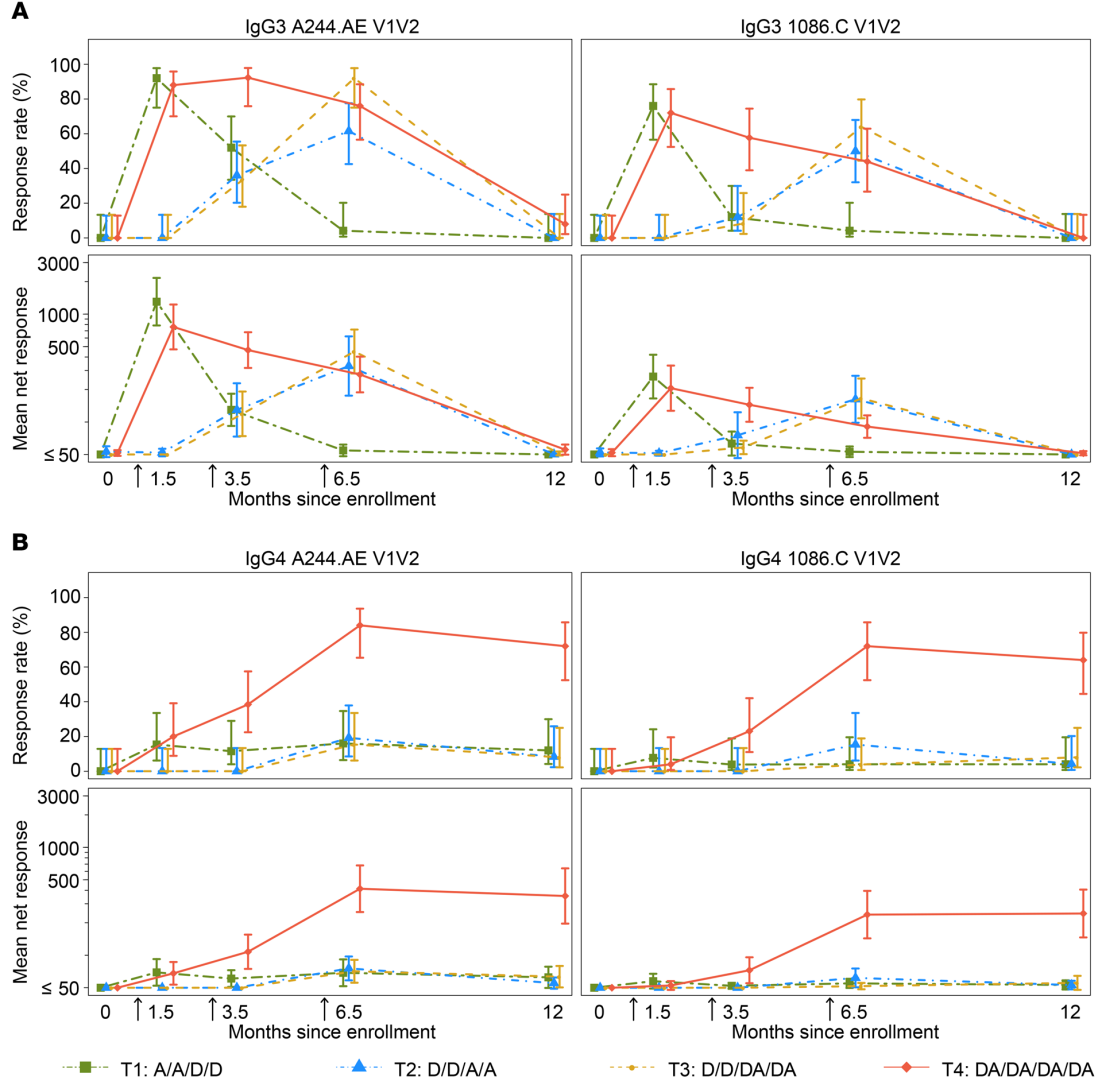
IgG3 and IgG4 binding-antibody responses in HVTN 105 participants over time, as measured by binding-antibody multiplex assay (BAMA) against 2 V1V2 antigens. Shown are the positive-response rates and 95% CI for IgG3 (**A**) and IgG4 (**B**) estimated using the score test method (top panels) and the geometric mean response magnitudes among all participants and 95% CI based on an assumption of log(IgG) following a normal distribution (bottom panels) by time point and treatment group (*n* = 25, 26, 26, 25 in T1–T4, respectively). The lines connect the response rates and geometric mean magnitudes between time points. Subtype AE V1V2: A244.AE V1V2; subtype C V1V2: 1086.C V1V2. Arrows indicate the second, third, and fourth vaccinations. D, DNA; A, AIDSVAX B/E.

Assessment of IgG3 and IgG4 subclass binding to HIV Env and V1V2 was also conducted longitudinally for all treatment groups. At month 6.5 (2 weeks after the fourth vaccination), IgG3 binding to A244.AE V1V2 was observed in the majority of participants in T2, T3, and T4 (62%, 92%, and 76%, respectively), with similar findings for the 1086.C V1V2 antigen in these 3 groups (50%, 64%, and 44%, respectively) (Figure 4A). Of note, peak IgG3 binding responses were observed 2 weeks after the second vaccination with AIDSVAX B/E, with no significant differences in response rates or magnitude between T3 and T4. Similarly, T1 participants had peak IgG3 responses similar in frequency and magnitude to those of T4 at month 1.5, but these responses rapidly waned to become negligible by month 6.5 (2 weeks after the second boost with DNA-HIV alone). Durability of IgG3 binding responses at month 12 (6 months after the fourth vaccination) was poor, with extremely low or absent responses across all antigens in all treatment groups (Figure 4A and Supplemental Figure 3A). HIV-specific IgG4 responses in groups T1–T3 were low after each vaccination (Figure 4B). In contrast to groups T1–T3, in T4, HIV-specific IgG4 responses were significantly boosted after the third and fourth vaccination. Specifically, the rates of IgG4 response to the A244.AE V1V2 and 1086.C V1V2 antigens at month 6.5 were 84% and 72%, respectively, among participants in T4, as compared with a range of 15%–19% and 4%–15%, respectively, for these antigens in groups T1, T2, and T3. Also of note, unlike IgG3 responses, IgG4 responses among T4 participants persisted at month 12 (6 months after the fourth vaccination), without a significant decline in response rate or magnitude (Figure 4B and Supplemental Figure 3B).

**Figure 5:**
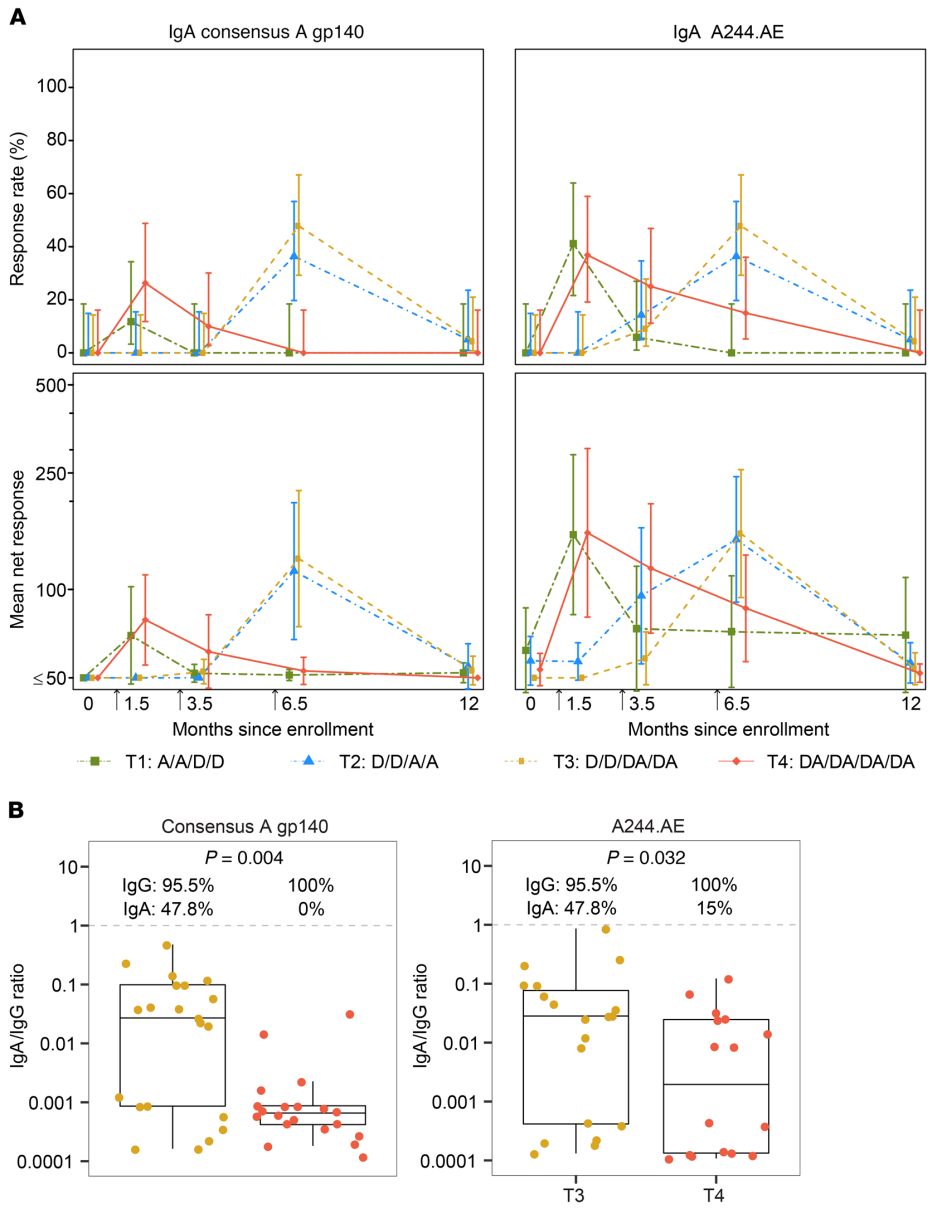
IgA binding-antibody responses in HVTN 105 participants over time and IgA/IgG ratio 2 weeks after the fourth vaccination, as measured by binding-antibody multiplex assay (BAMA) against consensus A gp140 and A244.AE antigens. (**A**) Shown are the positive-response rates and 95% CI estimated using the score test method (top panels) and the geometric mean response magnitudes among all participants and 95% CI based on an assumption of log(IgA) following a normal distribution (bottom panel) by time point and treatment group (*n* = 25, 26, 26, 25 in T1–T4, respectively). Arrows indicate the second, third, and fourth vaccinations. (**B**) The distribution and box-and-whisker plot of the IgA/IgG ratio in T3 and T4 (the midline of the box-and-whisker plot denotes the median and the ends of the box-and-whisker plot denote the 25th and 75th percentiles). Shown at the top of plots are the percentages of positive responders to IgA and IgG, respectively, in T3 and T4; and the *P* value is the testing difference in IgA/IgG ratio between T3 and T4 from Wilcoxon’s rank-sum test. D, DNA; A, AIDSVAX B/E.

Serum IgA binding responses to 2 HIV envelope proteins, consensus A gp140 (a direct correlate of risk identified in RV144; refs. 5, 8) and A244.AE (an AIDSVAX B/E–matched gp120), were examined longitudinally for all treatment groups (Figure 5). Similarly to IgG, IgA responses for each group peaked 2 weeks after administration of the second protein vaccination (at month 1.5 in T1 and T4, and at month 6.5 in T2 and T3). However, IgA responses were positive in less than 50% of participants in each group. At month 6.5 (2 weeks after the fourth vaccination), the IgA response rate was significantly higher in T3 than T4 for consensus A gp140 and A244.AE (Figure 5A), indicating that serum IgA responses were not boosted by the third and fourth doses of AIDSVAX B/E in T4. The IgA/IgG ratio at this time point was low for both groups, but statistically higher for T3 compared with T4 for the A244.AE antigen (Figure 5B). All groups had negligible IgA responses at month 12 (6 months after the fourth vaccination) (Figure 5A).

#### ADCC.

**Figure 6:**
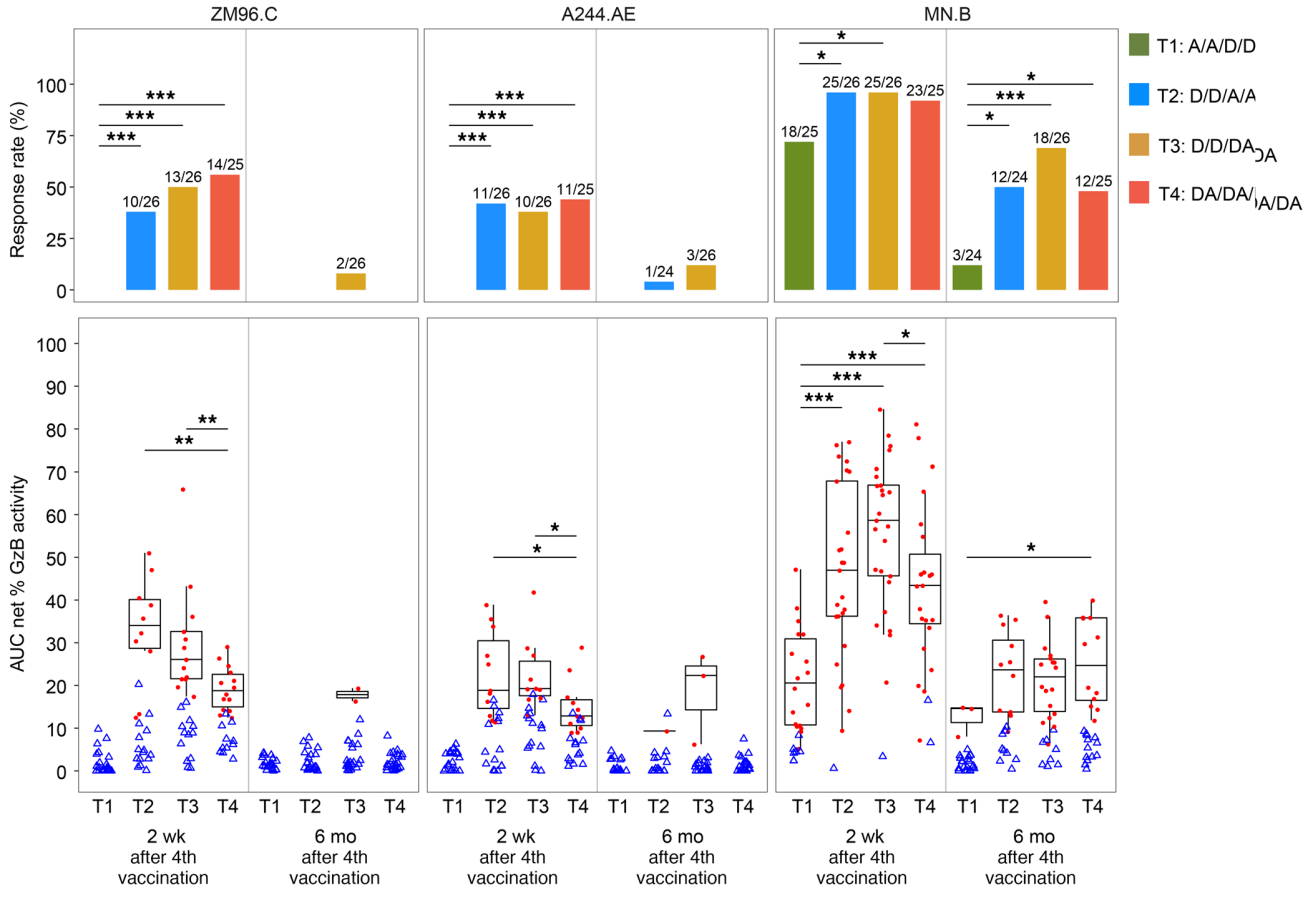
Antibody-dependent cell-mediated cytotoxicity (ADCC) at 2 weeks and 6 months after the fourth vaccination in HVTN 105. Shown are the response rates (top panels) and the distribution of AUC of granzyme B (GzB) activity (positive responders to peak granzyme B activity in red circles and nonresponders in blue triangles) and the box-and-whisker plots among the positive responders to peak granzyme B activity (the midline of the box-and-whisker plot denotes the median and the ends of the box-and-whisker plot denote the 25th and 75th percentiles) (bottom panels) by time point and treatment group (*n* = 25, 26, 26, 25 in T1–T4, respectively). Subtype C gp120: ZM96.C; subtype AE gp120: A244.AE; and subtype B gp120: MN.B. Bars and asterisks presented on the top of plots indicate the significant differences between treatment groups within the same visit (*P ≤ 0.05; **P ≤ 0.01; ***P ≤ 0.001) without multiple-comparisons adjustment. The comparisons between treatment groups were performed using Fisher’s exact test for response rates and Wilcoxon’s rank-sum test for magnitudes. Fractions above bars on the top panels indicate numbers of positive responses over the total numbers of responses (positive and negative) by time point and treatment group. D, DNA; A, AIDSVAX B/E.

Assessment of ADCC, as measured by the GranToxiLux (GTL) assay, was performed at the month 6.5 and month 12 time points (2 weeks and 6 months after the fourth vaccination, respectively) for all treatment groups. ADCC-GTL responses were observed for the 3 vaccine-matched gp120 antigens in T2, T3, and T4 at month 6.5 (Figure 6). Responses to the subtype B antigen, MN.B, in these groups were present in over 90% of participants, and responses persisted at month 12 in about half of the participants. ADCC-GTL responses to the ZM96.C-coated (subtype C) and A244.AE-coated (subtype A/E) targets were detected in a similar proportion of T2, T3, and T4 participants (ranging between 38% and 56%) at the month 6.5 time point; however, negligible responses were observed by month 12. Finally, ADCC-GTL responses among T1 group participants were detected only against the subtype B antigen, MN.B, but were significantly less frequent compared with the other 3 groups for this antigen by month 12. The magnitude of ADCC responses measured as area under the curve (AUC) recapitulated the response rate. The highest magnitudes were observed against the MN.B-coated targets in all 4 groups at the month 6.5 and 12 time points, and the AUCs were significantly higher in T3 compared with T4 two weeks, but not 6 months, after the fourth immunization.

#### Neutralizing-antibody responses.

**Figure 7:**
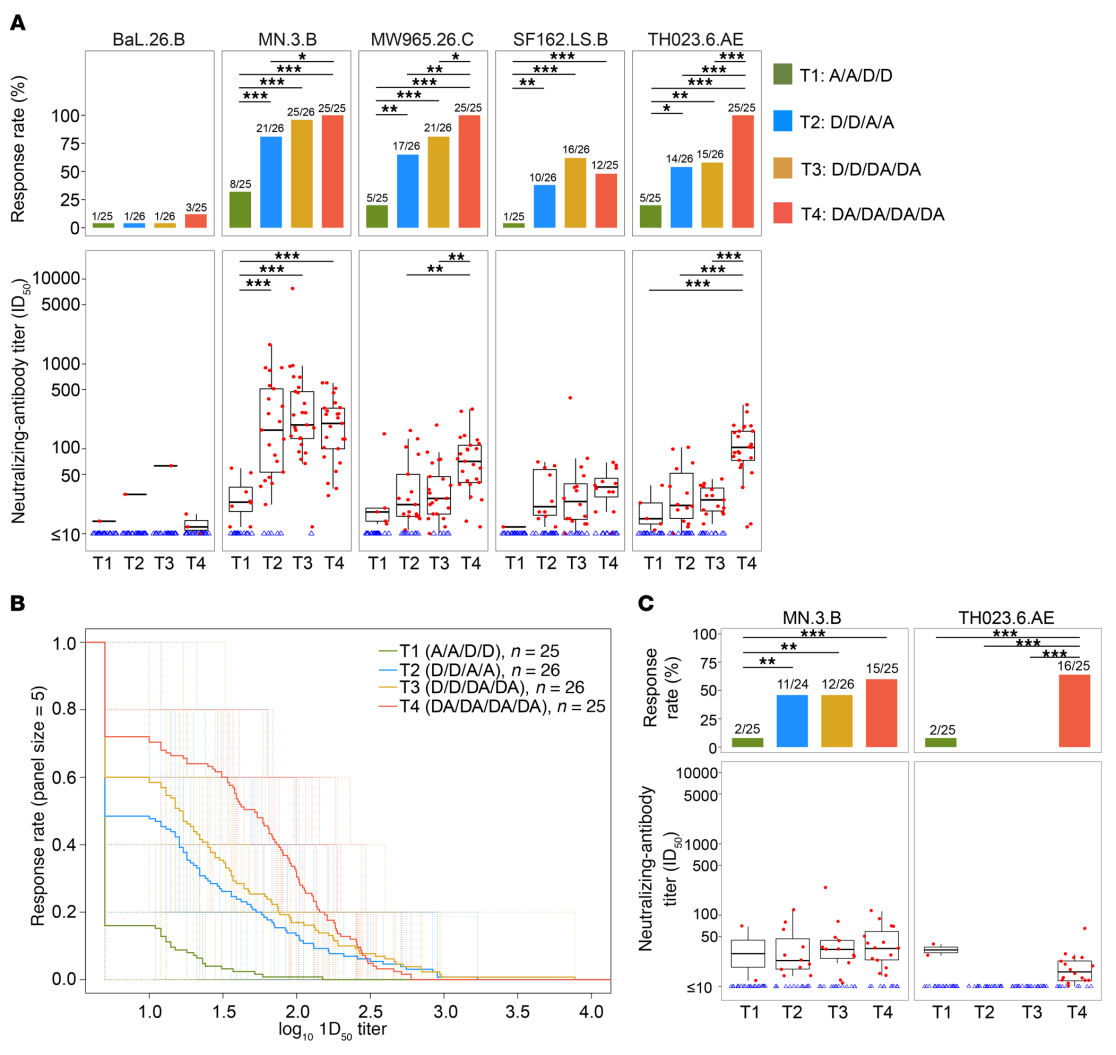
Neutralizing-antibody responses in HVTN 105. (**A**) Tier 1 Env-pseudotyped viruses (BaL.26.B, MN.3.B, MW965.26.C, SF162.LS.B, and TH023.6.AE) were tested in the TZM-bl neutralization assay 2 weeks after the fourth vaccination (peak time point). Bar plots show positive-response rates by treatment group on top panels. The bottom panels show the distribution of response titer (positive responses in filled red circles and negative responses in open blue triangles) and the box-and-whisker plots of response titer among positive responders (the midline of the box-and-whisker plot denotes the median and the ends of the box-and-whisker plot denote the 25th and 75th percentiles) by treatment group (*n* = 25, 26, 26, 25 in T1-T4, respectively). (**B**) Neutralizing-antibody magnitude breadth (AUC-MB) curves for TZM-bl based on all 5 isolates. (**C**) Neutralizing-antibody responses to MN.3.B and TH023.6.AE at 6 months after the fourth vaccination. Bars and asterisks presented on the top of plots in **A** and **C** indicate the significant differences between treatment groups within the same visit (*P ≤ 0.05; **P ≤ 0.01; ***P ≤ 0.001) without multiple-comparisons adjustment. The comparisons between treatment groups were performed using Fisher’s exact test for response rates and Wilcoxon’s rank-sum test for magnitudes. The comparisons of AUC-MB at peak time point in **B** between treatment groups from Wilcoxon’s rank-sum test show that AUC-MB in T4 is significantly higher than T1–T3, with *P* < 0.001, *P* < 0.001, and *P* < 0.003, respectively, and AUC-MB in T1 is significantly lower than T2–T3, with *P* < 0.001. Fractions above bars in **A** and **C** indicate the numbers of positive responders over the total number of responses (positive and negative). D, DNA; A, AIDSVAX B/E.

Neutralizing-antibody (nAb) response assays to tier 1 HIV strains were assessed at the month 6.5 and month 12 time points (2 weeks and 6 months after the fourth vaccination, respectively) for all treatment groups. At month 6.5, response rates and magnitudes varied among the 5 tier 1 strains, but tended to be highest for those more closely matched to the vaccine antigens MN.3.B (subtype B), MW965.26.C (subtype C), and TH023.6.AE (subtype A/E) (Figure 7A). Participants in T1 had significantly lower nAb response rates and magnitudes than participants in the other groups. Participants in T4 had the strongest responses of the 4 treatment groups for MW965.26.C and TH023.6.AE. Of note, peak rates of nAb responses to strains MW965.26.C and TH023.6.AE were significantly higher in T4 compared with T3 (*P* = 0.05 and 0.0003, respectively) and T2 (*P* = 0.002 and <0.0001, respectively). Also, the nAb titers for these 2 strains were significantly higher in T4 compared with T3 or T2 at this time point for the MW965.26.C (T4 vs. T3: *P* = 0.002; T4 vs. T2: *P* = 0.008) and for the TH023.6.C strain (T4 vs. T3: *P* < 0.0001; T4 vs. T2: *P* = 0.0001). Similarly, T4 performed best in the neutralization magnitude-breadth (MB) analysis, with a significantly higher AUC-MB than T2 and T3 (*P* = 0.0004 and 0.003, respectively) (Figure 7B). At month 12 (6 months after the fourth vaccination), nAb responses among all groups had declined; however, neutralization of MN.3.B was still present in groups T2, T3, and T4 (45.8%, 46.2%, and 60%, respectively) (Figure 7C). Of note, the response to TH023.6.AE was detected at month 12 in T4 (64%) and not in T2 and T3.

### Vaccine-induced cellular immunogenicity

**Figure 8:**
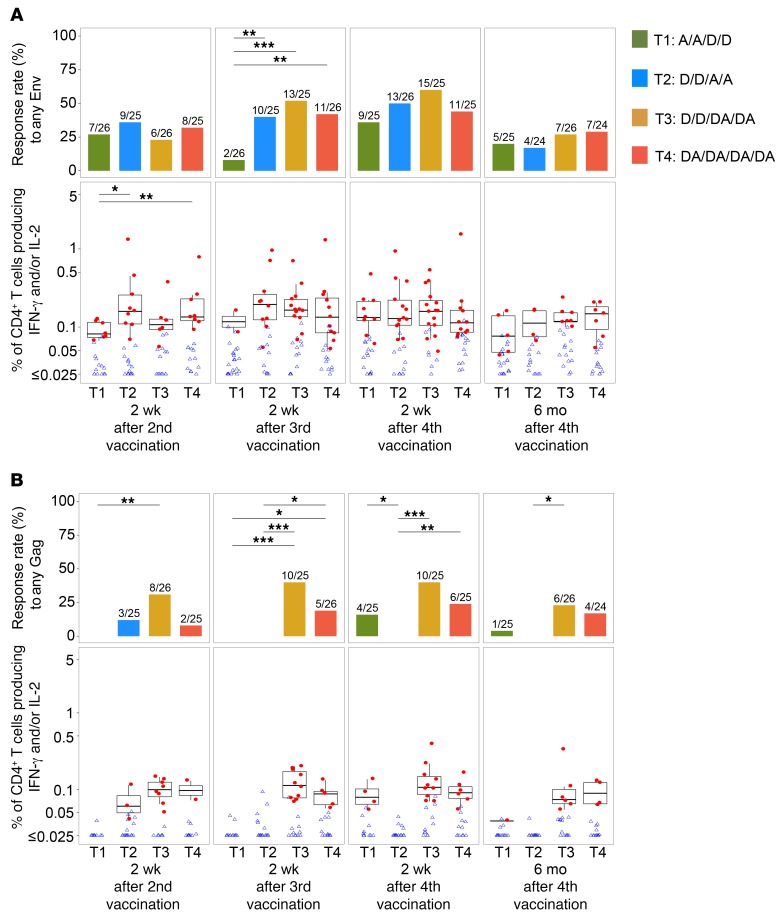
CD4^+^ T cell responses elicited in HVTN 105, as measured by intracellular cytokine staining (ICS), and reported as the percentage of cells producing IFN-γ and/or IL-2 in each treatment group. (**A**) CD4^+^ T cell responses to any HIV Env peptide pools (Any Env), all vaccine-matched: ZM96 gp140-Env1, ZM96 gp140-Env2, and 92TH023-Env. (**B**) CD4^+^ T cell responses to HIV Gag peptide pool (Any Gag): ZM96 Gag. Bar plots on top panels show the positive-response rates by time point and treatment group (*n* = 25, 26, 25, 25 in T1–T4, respectively). The bottom panels show the distribution of response magnitudes (positive responses in filled red circles and negative responses in open blue triangles) and the box-and-whisker plots of magnitudes among the positive responders (the midline of the box-and-whisker plot denotes the median and the ends of the box-and-whisker plot denote the 25th and 75th percentiles). Fractions above bars on top panels indicate the numbers of positive responders over the total numbers of responses (positive and negative). Bars and asterisks on top of plots indicate the significant differences between treatment groups (*P ≤ 0.05; **P ≤ 0.01; ***P ≤ 0.001) without multiple-comparisons adjustment. The comparisons between treatment groups were performed using Fisher’s exact test for response rates and Wilcoxon’s rank-sum test for magnitudes. D, DNA; A, AIDSVAX B/E.

Positivity of responses for CD4^+^ T cells expressing IL-2 and/or IFN-γ was determined 2 weeks after the second, third, and fourth vaccination time points and 6 months after the fourth vaccination. At 2 weeks after the fourth vaccination, the CD4^+^ T cell response rate to Env ranged from 36% to 60% among the 4 groups, with the highest response rate in T3, but the differences did not reach statistical significance (Figure 8A). At the month 12 time point (6 months after the fourth vaccination), CD4^+^ responses to Env declined to 17%–29% but again without significant differences between groups. CD4^+^ T cell responses to Gag, which reflected immune responses to the DNA vaccine alone, were generally less frequent and of lower magnitude compared with Env (Figure 8B). Overall, there were no statistically significant differences in the rate or magnitude of CD4^+^ T cell responses to Gag between T3 and T4 at any time point, although there was a trend for higher responses in T3. Finally, there were negligible CD8^+^ T cell responses to Env or Gag, as measured by IL-2 and IFN-γ, in any of the study groups (Supplemental Figure 4, A and B).

**Figure 9:**
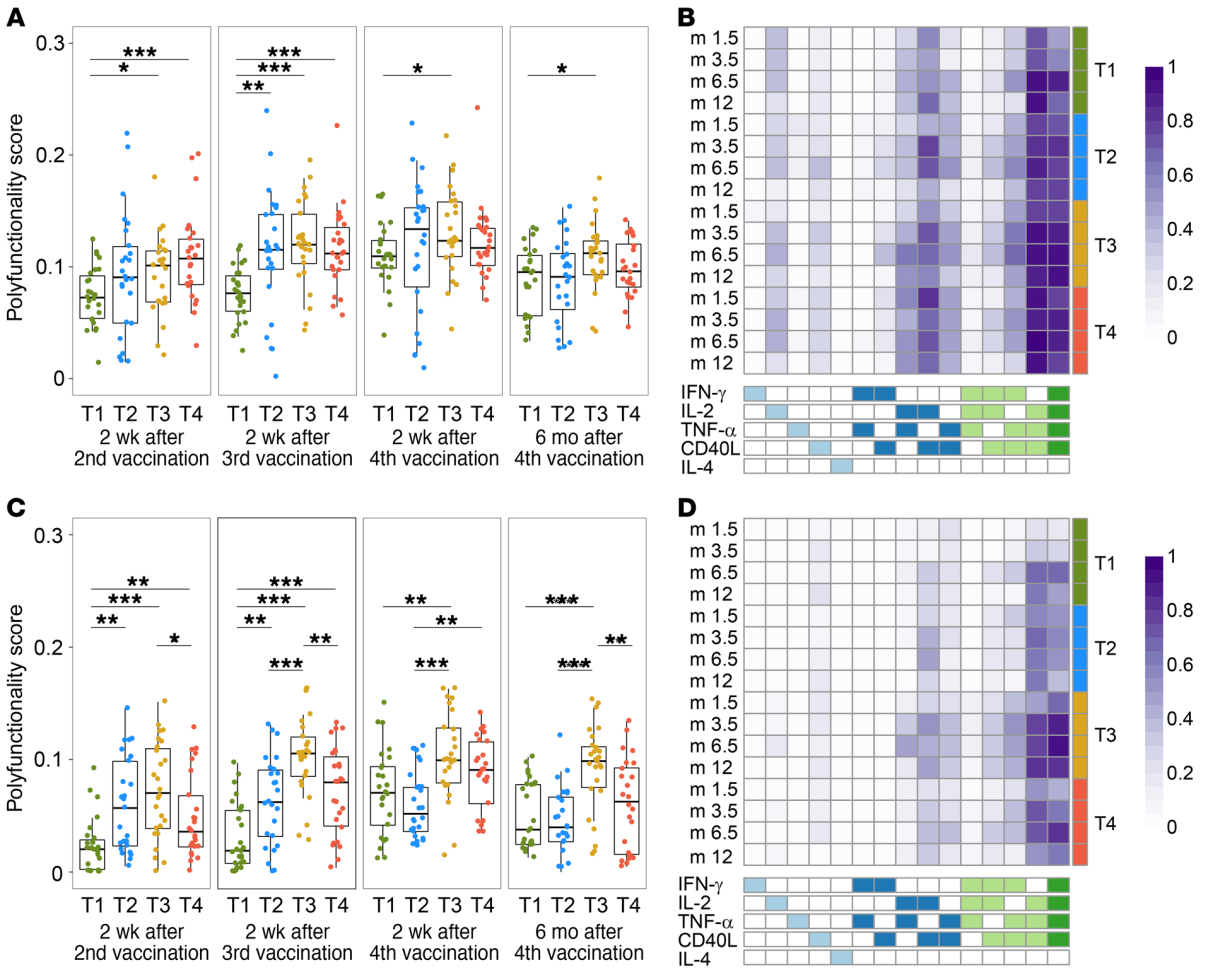
COMPASS CD4^+^ T cell polyfunctionality (PF) scores and mean probability of response heatmaps. The distribution and the box-and-whisker plot of PF scores for all vaccine-matched HIV Env peptide pools: ZM96 gp140-Env1, ZM96 gp140-Env2, and 92TH023-Env (**A**) and for ZM96 Gag peptide pool (**C**) by time point and treatment group (*n* = 25, 26, 25, 25 in T1–T4, respectively). The midline of the box-and-whisker plot denotes the median and the ends of the box-and-whisker plot denote the 25th and 75th percentiles. Bars and asterisks on top of box-and-whisker plots indicate the significant differences between treatment groups using Wilcoxon’s rank-sum test (*P ≤ 0.05; **P ≤ 0.01; ***P ≤ 0.001) without multiple-comparisons adjustment. Heatmaps for CD4^+^ T cell response to any Env (**B**) and ZM96 Gag (**D**) show the mean posterior probabilities of antigen-specific responses from COMPASS. Columns correspond to the different subsets of cytokines being considered and rows correspond to mean across the individual participants in each treatment group at each time point. Each cell shows the probability that the corresponding antigen-specific subset (column) is being expressed in the corresponding treatment group in average (row), and is color coded ranging from white (zero) to dark purple (one).

CD4^+^ T cell polyfunctionality (PF) scores for Env (Figure 9A) and Gag (Figure 9C) peptides were assessed at the 4 time points using combinatorial PF analysis of single cells (COMPASS). Env-specific CD4^+^ T cell PF scores were similar across all groups after the fourth vaccination, although somewhat lower for T1 and T4 (Figure 9A). PF scores for Gag again indicated that at least 2 doses of DNA were required for optimal responses, and responses in T3 and T4 peaked 2 weeks after the third vaccination, with the PF scores for T3 significantly higher than for T4 at that time point (*P* = 0.007), as well as at 6 months after the fourth vaccination (*P* = 0.005).

There were 2 dominant polyfunctional populations among Env-specific CD4^+^ T cell responses, one identified by 4 markers (IFN-γ^+^IL-2^+^TNF-α^+^CD40L^+^) and the second with 3 markers (IL-2^+^TNF-α^+^CD40L^+^ but without IFN-γ) (Figure 9B). A high mean probability of response for both of these populations across all groups at most time points indicates that many, and often most, individuals had detectable CD4^+^ T cells expressing these markers above the level in the negative control stimulation. Cells expressing only IL-2 and IL-2 in combination with CD40L were detected in some individuals, and there were minimal differences between groups. The other commonly expressed subsets included cells coexpressing TNF-α and CD40L, with or without IFN-γ. Compared with Env, the same two 3- and 4-function subsets were dominant for Gag (Figure 9D), with highest expression of these subsets in T3, likely accounting for the significantly higher PF score compared with the other groups even though T3 only received 2 doses of Gag compared with the 4 doses that T4 received. A few of the other double- and triple-positive cells observed for Env were also detected in more individuals in T3 for Gag. These results highlight the induction of highly polyfunctional Env- and Gag-specific CD4^+^ T cells, and are also in agreement with the highest IFN-γ and/or IL-2 response rates for T3 (although not statistically significant) compared with the other groups.

### Overview of the induced immune responses

**Figure 10:**
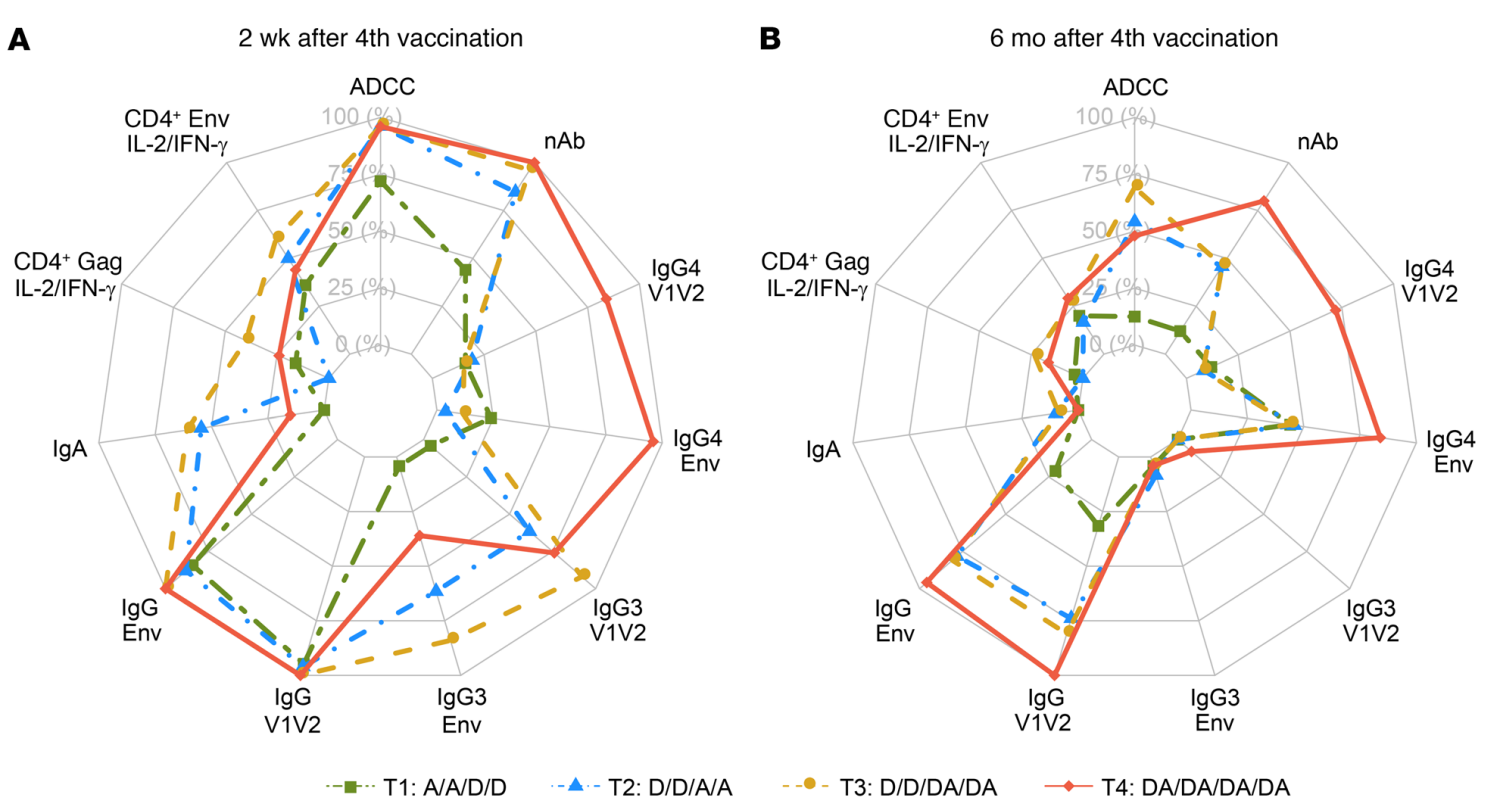
Radar plots. Maximum response rates of each assay readout at 2 weeks (**A**) and 6 months (**B**) after the fourth vaccination. Antigens included in each assay readout are as follows: For IgG binding-antibody responses to Env, antigens include A244.AE, MN.B, ZM96.C, Con S gp140 CFI, as well as Con 6 gp120. For IgG binding-antibody responses to V1V2, antigens include 1086.C V1V2, CaseA2_gp70_V1V2.B, CaseA2_V1/V2/169K.B, A244.AE V1V2, as well as ZM96.C V1V2. For IgA binding-antibody responses, antigens include consensus A gp140 and A244.AE. For neutralizing-antibody (nAb) responses, antigens include BaL.26.B, MN.3.B, MW965.26.C, SF162.LS.B, and TH023.6.AE. For antibody-dependent cell-mediated cytotoxicity (ADCC) responses, antigens include ZM96.C, A244.AE, and MN.B. For CD4^+^ T cell intracellular cytokine staining, Env antigens are ZM96 or 92TH023 peptide pools (Any Env), and the Gag antigen is ZM96 peptide pool (Any Gag). D, DNA; A, AIDSVAX B/E.

A global representation of the distribution of the cellular and humoral immunogenicity by response rate is shown in Figure 10. The analysis indicates that coadministration of DNA and protein (T3 and T4 groups) can improve the response rates for most of the immune responses measured and induce a favorable immune profile at later time points. Suboptimal responses were seen when protein served as a prime for subsequent boosting with DNA (T1), which was particularly evident at the month 12 time point (6 months after the fourth vaccination). While priming with DNA and boosting with protein alone (T2) was immunogenic, the response was less favorable than the coadministration groups for IgA, IgG3, IgG4, and CD4^+^ Gag responses at month 6.5 (2 weeks after the fourth vaccination), and for ADCC, nAb, IgG4, and CD4 Gag at month 12 (6 months after the fourth vaccination).

### Vaccine-induced sero-reactivity was uncommon

Vaccine-induced sero-reactivity (VISR) was assessed by 4 licensed diagnostic kits used to detect HIV antibodies at 4 time points during the study. VISR was detected by all of the 4 assays in 4%–122% of participants in each group (3 participants in T4 and in 1 participant in each of the other 3 groups). None of the participants in the study became infected with HIV (Supplemental Table 2).

## Discussion

This trial evaluated different combinations of DNA (DNA-HIV-PT123) and protein (AIDSVAX B/E) vaccinations to determine which strategy would best elicit favorable HIV-specific antibody and T cell responses, as defined by the correlates of risk identified in RV144, over the 12-month duration of protocol follow-up. Our study showed that coadministration of DNA and protein from the initial vaccination (T4) leads to induction of potentially protective anti-HIV antibody responses within the first 6 weeks, as opposed to the DNA prime–protein boost regimen reflecting the RV144 vaccination schedule (T3), which induces similar responses, but not until after the final boosts at month 6. Otherwise, there were no significant differences between the early and delayed coadministration groups (T4 vs. T3) for induction of any of the currently known RV144 potential correlates of HIV acquisition risk. Of note, we observed a significant increase in HIV-specific IgG4 at the month 12 time point in the early coadministration group (T4), although the functional significance of HIV-specific IgG4 in the context of conferred protection from infection is currently unclear and subsequently discussed herein. The regimen with DNA priming administered at month 0 and 1 with AIDSVAX B/E boosting alone at months 3 and 6 (T2) induced immune responses similar to those of T3, but 4 doses of DNA (comparing T3 vs. T2) were superior in terms of inducing a better CD4^+^ T cell responses to Gag, which was contained only in the DNA candidate. Finally, priming with protein and boosting with DNA (T1) clearly led to inferior immunologic results compared with the other groups at the month 6 time point and beyond.

In this proof-of-concept study, we demonstrated that substituting DNA for the ALVAC vector (T3) is a suitable alternative. Interestingly, the prime-boost regimen in our study was clade mismatched relative to RV144 (DNA plasmid expressed a subtype C Env rather than the clade-matched AE Env in RV144), raising the possibility that subtype C Env can serve as a universal prime for heterologous Env protein boosts. In fact, comparing data from this group to RV144 (5, 20), the peak BAMA IgG response rate 2 weeks and 6 months after the final vaccination (months 6.5 and 12) was 96%–100% and 48%–81%, respectively, against the 2 strains (subtype B MN gp120 and subtype A/E A244 gp120) contained in AIDSVAX B/E, the vaccine common to both HVTN 105 and RV144. Similarly, IgG responses to the V1V2 scaffold of subtype C 1086.C V1V2, which was identified as an inverse correlate of risk in RV144, were present at months 6.5 and 12 in 100% and 67% of participants in T3, respectively. Of note, the magnitude of the IgG response to the V1V2 scaffold was quite high in T3 (peak geometric mean titer [GMT] to 1086.C V1V2 in T3 was 15,002 [95% CI: 10,099–22,285]). Importantly, responses in our study were more durable than in RV144, persisting at month 12 (6 months after the fourth vaccination) in up to 40% of T3 recipients versus 10% in RV144 vaccine recipients to 1086.C V1V2 antigen, although an even greater response at month 12 would be desirable. By extension, the coadministration of DNA and protein at all vaccination time points (T4) also compares favorably to RV144. This is especially evident when considering the rapid induction of antibodies, but also in terms of sustained responses. All participants in T4 had remarkably elevated IgG response to 1086.C V1V2 antigen (100% response rate and GMT = 14,809 at 6.5 months), with 71% of vaccinees having detectable antibody response at 12 months from first vaccination.

In RV144, IgG3 responses against V1V2 were associated with low risk of HIV-1 acquisition, and postulated to mediate protective HIV-specific antibody Fc effector functions such as ADCC (7) and antibody-dependent cell-mediated phagocytosis (21, 22). There is also evidence from experiments with monoclonal antibodies derived from B cells of RV144 vaccinees that HIV-1 Env IgA blocks ADCC-mediating antibodies, consistent with the findings that ADCC correlated with decreased risk of HIV-1 infection when Env IgA was low and that an elevated IgA/IgG ratio correlated with HIV-1 risk (8). In our study, high peak net percentage granzyme B ADCC activity and response rates were noted for groups T2, T3, and T4 against subtype B gp120 and remained elevated at the month 12 time point (6 months after the fourth vaccination). As seen in RV144 (20), the IgG3 responses in HVTN 105 significantly declined over time, although the persisting ADCC activity at month 12 could be explained either by the favorable low IgA/IgG ratio at the completion of vaccine regimen dosing (especially seen in T4) and/or by the overall persistence of other IgG subclasses such as IgG1 or alteration of IgG1 glycosylation mediating ADCC at the durability time point, as demonstrated through a systems serology approach (23).

In our trial, vaccination with 4 protein immunizations (T4) elicited greater Env-specific IgG4 responses than the other immunization groups, although this is more likely explained by the administration of the AIDSVAX B/E protein at 4 time points over the 6-month vaccination schedule as opposed to coadministration with DNA, per se. Bias toward the induction of the HIV-specific IgG4 subclass has been seen in other studies of repeated vaccination with AIDSVAX B/E (24, 25), including in VAX003, which administered AIDSVAX B/E alone at 7 time points to injection-drug users over a 3-year follow-up (3), and in RV307, which administered 2 additional doses of AIDVAX B/E to RV144 participants 6–8 years after previous vaccination (26). This phenomenon has also been noted with acellular pertussis vaccine after repeated vaccination in a pediatric population. However, in a follow-up study in children infected with *Bordetella pertussis*, the IgG-subclass distribution did not differ from healthy vaccinated children, suggesting the absence of clinical importance (27). Therefore, the significance of high levels of durable Env-specific IgG4 and its correlation with existing in vitro ADCC assays in the setting of HIV acquisition is unclear and should be further examined in ongoing HIV vaccine efficacy trials.

The CD4^+^ T cell responses were similar across all the groups after the fourth vaccination. This is especially surprising for Gag since that was only encoded for in the DNA, and this difference in response rates may indicate some interference or antigenic competition due to the additional doses of Env protein. Consistent with the IL-2 and/or IFN-γ response rates, the Gag-specific PF scores were also lower for the group with 4 protein vaccinations. Overall, CD4^+^ T cell responses included multiple 2-, 3-, and 4-function subsets, mainly including CD40L as one of the functions. This suggests that a major function for these vaccine-induced T cells is to provide help to B cells. It may be surprising that IL-4 was not detected among these responding cells, and this could be due to low sensitivity for detection of IL-4 in our assay or in fact due to poor induction of T helper type 2 (Th2) cells that may be necessary for a more durable antibody response. It would be potentially of benefit to also induce CD8^+^ T cells in any candidate HIV regimen. Although the regimens we tested did not induce CD8^+^ T cells, alternate DNA vaccines, especially when administered with electroporation, are now capable of inducing CD8^+^ T cells and may be appropriate substitutions for the DNA part of the vaccine regimen (28–30).

The type of prime as well as the combination and sequence in which vaccines are administered influence the quality of immune responses. Administering a protein-only prime (T1) did not elicit strong, durable antibody responses in this study. It is possible that with a more potent adjuvant, as was used in the preclinical studies in which a protein prime did elicit strong responses, different results may be observed. The group with DNA-only prime followed by protein-only boost (T2) did elicit antibody responses, but these were generally lower in response rate and magnitude than the groups that included coadministration of DNA and protein. The 2 groups that received DNA and protein coadministration at the last 2 (T3) or at all 4 (T4) vaccination time points elicited not only high V1V2 IgG and antibody effector function (ADCC) responses, but also tier 1 nAb and CD4^+^ T cell responses, higher or similar to those observed in RV144. Based on our data, it is not possible to make a firm conclusion about whether T3 versus T4 is the preferred regimen, as the potential correlates of risk identified in RV144 have not yet been confirmed. T4 induced early responses requiring, however, more vaccine doses. Both T3 and T4 induced equivalent cellular and humoral responses, with the exception of a marked increase in IgG4 with subsequent protein boost in T4; the physiologic significance of that increase is yet to be determined. Future studies will examine other potential correlates of protective immunity, such as avidity of IgG antibodies and other antibody Fc effector functions.

While no DNA vaccine is licensed for use in humans, many DNA vaccines are approved for veterinary applications. DNA vaccines are currently widely evaluated in clinical trials against many infectious diseases (31, 32) and for HIV prevention and therapeutics. Ongoing trials are testing the protein and DNA combination regimens with alternative adjuvants given with envelope protein (e.g., MF59 or AS01) (HVTN 108 [NCT02915016] and HVTN 111 [NCT02997969]) (33), modification of the schedule of envelope protein administration, or coadministration with other vaccine platforms such as adenoviral vector 26 or MVA. There are also other approaches to optimize DNA priming, such as formulation with cytokine adjuvants or administration by intramuscular or intradermal electroporation, that can also be examined to expand our understanding of tailoring an optimal immunologic signature to prevent acquisition of HIV. Current studies are testing ALVAC as an HIV vaccine candidate including a pivotal phase 2b/3 trial in South Africa (HVTN 702 [NCT02968849]) in addition to early-phase studies (HVTN 107 [NCT NCT03284710] and HVTN 120 [NCT03122223]). DNA vaccines are safe, easy to manufacture, and have great molecular stability and flexibility with immunogen design. Based on our study, DNA provides a suitable alternative platform to ALVAC; however, a larger efficacy study is needed to confirm the findings of our proof of concept.

In conclusion, DNA appears to be a good alternative to viral vectors, and its early coadministration with protein can rapidly induce a potentially protective antibody response that would enhance its value to public health.

## Methods

### Trial design.

The objectives of the study were to evaluate the safety and tolerability (primary objective) and the immunogenicity (secondary objective) of the combination of AIDSVAX B/E and DNA-HIV-PT123 administered in different sequences or simultaneously in HIV-uninfected healthy adults.

Participants were screened at 7 HVTN sites in 6 US cities: Nashville, Tennessee; New York, New York; Rochester, New York; Philadelphia, Pennsylvania; Seattle, Washington; and San Francisco, California. Participants were males and nonpregnant females who met the inclusion criteria as listed on https://clinicaltrials.gov Women of child-bearing potential were advised to avoid pregnancy for 12 months after enrollment. All participants were assessed to be at low risk for HIV acquisition, were counseled routinely about HIV risk reduction, and were assessed for potential social impacts of study participation. Figure 1 ​shows a CONSORT statement flow chart of study enrollment, allocation, and safety analysis.

Participants were randomly assigned to 1 of 4 groups with an allocation ratio of 1:1:1:1 (Table 1). Participants received different combinations of AIDSVAX B/E, DNA-HIV-PT123, and placebo, administered intramuscularly. AIDSVAX B/E consisted of 300 μg of subtype B (MN) HIV gp120 glycoprotein and 300 μg of subtype A/E (A244) HIV gp120 glycoprotein adsorbed onto aluminum hydroxide gel adjuvant and administered into the right deltoid muscle. DNA-HIV-PT123 contained a mixture of 3 DNA plasmids: (a) clade C ZM96 *gag*, (b) clade C ZM96 *gp140*, and (c) clade C CN54 *pol-nef*, delivered at a total dose of 4 mg administered into the left deltoid muscle via needle and syringe. The placebo consisted of sodium chloride for injection, 0.9% USP. The randomization sequence was obtained by computer-generated random numbers and provided to the site by a central data monitoring center. A pharmacist at each site was responsible for ensuring the security of the randomization code and maintaining the anonymity of the sample by covering the syringe containing the study products. All participants and study staff, apart from the study pharmacist, were blinded to treatment assignment.

### Safety assessment.

Reactogenicity assessments were performed on all participants for 3 days following each injection. Participants recorded symptoms using a postvaccination symptom log and were contacted daily by the study site during each reactogenicity assessment period. Participants returned to the clinic 2 weeks after each vaccination and 9 months after the first vaccination for clinical evaluation for local and systemic signs and symptoms and laboratory testing of hematologic, renal, and hepatic analytes. These safety evaluations were coded according to the Medical Dictionary for Regulatory Activities terminology, and graded using the US Division of AIDS Table for Grading the Severity of Adult and Pediatric Adverse Events (34, 35). AEs were assessed for their relationship to the study product. Reactogenicity symptoms among the treatment groups were tabulated by severity.

### Sample processing.

Serum for humoral assays was obtained from serum-separating tubes (SSTs) and frozen at –80°C. Peripheral blood mononuclear cells (PBMCs) for cellular assays were isolated and cryopreserved from heparin-anticoagulated whole blood within 6 hours of venipuncture, as described previously (36).

### Binding-antibody assays.

Serum HIV-1–specific IgG, IgG3, IgG4, and IgA responses were measured with a custom HIV-1–binding antibody multiplex assay (BAMA) as previously described (7, 37) using gp120 proteins and V1V2 antigens detailed previously (38). Details of the antigens included are listed in Supplemental Table 3.

### ADCC.

ADCC-mediated antibody responses were measured by ADCC GranToxiLux (GTL) and tested against ZM96.C, A244.AE, and MN.B using gp120-coated cells (percentage granzyme B readout) (see Supplemental Table 3 for additional details on the antigens used). Participant sera were incubated with effector cells and gp120-coated target cells (39) and ADCC was quantified as net percentage granzyme B activity, which is the percentage of target cells positive for GTL detected by flow cytometry. For each participant at each time point, the percentage granzyme B activity was measured at 6 dilution levels: 1:50, 1:250, 1:1250, 1:6250, 1:31,250, and 1:156,250 for each antigen. For some participants, percentage granzyme B activity was measured at an additional 6-dilution series: 1:6250, 1:31,250, 1:156,250, 1:781,250, 1:3,906,250, and 1:19,531,250 for some antigens. The additional dilution series was used because the endpoint dilution for some of the samples could not be determined with the initial series. Overlapping dilutions between series were averaged. The analyses in the data focused on the following readouts: (a) peak net percentage granzyme B activity defined as the maximum activity across the 6 or 9 dilution levels (peak activity), and (b) nonparametric area under the net percentage granzyme B activity versus log_10_(dilution) curve (AUC), calculated using the trapezoidal rule. Peak activity less than 0% was set to 0%. A positive response was defined as peak activity greater than or equal to 8%.

### HIV-1–specific nAb assays.

nAbs against HIV-1 were measured as a function of reductions in Tat-regulated luciferase (Luc) reporter gene expression in TZM-bl cells 2 weeks and 6 months after the last vaccination (40). The assay measured neutralization titers against 2 Env-pseudotyped viruses that exhibit a tier 1 neutralization phenotype and are matched to the vaccine strains in AIDSVAX B/E (MN.3.B and TH023.6.AE), a panel of heterologous Env-pseudotyped viruses that exhibit a tier 1 neutralization phenotype (see Supplemental Table 3 for additional details on the antigens used) or a tier 2 neutralization phenotype (Global Panel: 246-F3_C10_2, 25710-2.43, 398-F1_F6_20, BJOX002000.03.2, Ce1176_A3, Ce703010217_B6, CH119.10, CNE55, CNE8, TRO.11, X1632-S2-B10, and X2278_C2_B6). Response to a virus/isolate in the TZM-bl assay is considered positive if the neutralization titer is above a prespecified cutoff (one-half the lowest dilution tested). A titer was defined as the serum dilution that reduces relative luminescence units (RLUs) by 50% relative to the RLUs in virus control wells (cells + virus only) after subtraction of background RLU (cells only). The prespecified cutoff was 10 for TZM-bl cells.

### Intracellular cytokine staining assay.

Flow cytometry was used to examine HIV-1–specific CD4^+^ and CD8^+^ T cell responses using a validated intracellular cytokine staining (ICS) assay 2 weeks after each boost as well as 12 months after enrollment. The peptide pools evaluated were vaccine matched (ZM96 gp140-Env1, ZM96 gp140-Env2, 92TH023-Env, and ZM96 Gag), covering Env and Gag (Supplemental Table 3). Previously cryopreserved PBMCs were stimulated with the synthetic peptide pools. As a negative control, cells were not stimulated. As a positive control, cells were stimulated with a polyclonal stimulant, phytohemagglutinin (PHA). There were no replicates except for the negative control, which has 2 replicates.

Responses of CD4^+^ and CD8^+^ T cells measured by ICS for IL-2 and/or IFN-γ to any HIV peptide pool (ZM96 gp140-Env 1, ZM96 gp140-Env 2, 92TH023-Env, and ZM96 Gag) are primary immunogenicity endpoints. ZM96 gp140-Env1 and -Env2 represent the N-terminal and C-terminal half of ZM96 gp140, respectively; since they were not overlapping, the response to ZM96 gp140 is represented as the sum of both pools. 92TH023-Env represents the full 92TH023 gp140; responses to “Any Env” are therefore represented as the maximum of ZM96 gp140-Env1 and -Env2 and 92TH023 Env. The response to “Any HIV protein” was defined as the sum of the response to Any Env and the response to Gag.

Several criteria were used to determine if data from an assay are acceptable and can be statistically analyzed. The blood draw date had to be within the allowable visit window as determined by the protocol. On the second day after sample thawing, the viability had to be 66% or greater. If not, the sample for that specimen at that time point had to be retested. If upon retesting the viability remained below this threshold, the ICS assay was not performed and no data were reported to the statistical center for that time point. For the negative control acceptance criteria, if the average cytokine response for the negative control wells was above 0.1% for either the CD4^+^ or CD8^+^ T cells, then the sample had to be retested; it was the response for the retested sample that was analyzed.

The total number of CD4^+^ and CD8^+^ T cells had to exceed certain thresholds. If the number of CD4^+^ or CD8^+^ T cells was less than 5,000 for any of the HIV-1 peptide pools or one of the negative control replicates for a particular sample, data for that stimulation were filtered. If both negative control replicates contained fewer than 5,000 cells, the sample was retested. If upon retesting one negative control replicate contained fewer than 5,000 cells, the negative control replicate with more than 5,000 cells was used. If both negative control replicates from the retest for a T cell subset had fewer than 5,000 cells, then the data for the T cell subset were not included in the analysis.

### T cell PF analyses.

COMPASS is a computational framework for unbiased PF analysis of antigen-specific T cell subsets (41). COMPASS uses a Bayesian hierarchical framework to model all observed functional cell subsets and select those most likely to exhibit antigen-specific responses. Cell-subset responses were quantified by posterior probabilities, while participant-level responses were quantified by 2 summary statistics (functionality scores) that can be correlated directly with outcomes of interest, and describe the quality of an individual’s functional response. The functionality score is defined as the estimated proportion of antigen-specific subsets detected among all possible ones. The PF score is similar, but it weighs the different subsets by their degree of functionality, naturally favoring subsets with higher degrees of functions, motivated by the observation that higher degree function has been correlated with good outcomes in certain vaccine studies. For this analysis, expression of IFN-γ, IL-2, TNF-α, IL-4, and CD40L was included. Scores were compared between treatment groups using Wilcoxon’s rank-sum test. For Env-specific PF scores, overall responses across pools were computed by summing the cell counts, both for the number of expressing cells and the total cell count (numerator and denominator), in fitting a COMPASS model. For Any Env, these were summed for all 3 Env peptide pools. A heatmap for each stimulation and T cell subset shows the mean posterior probabilities of antigen-specific responses from COMPASS. Columns correspond to the different subsets of cytokines being considered and rows correspond to the mean across the individual participants in each treatment group at each time point. Each cell shows the probability that the corresponding antigen-specific subset (column) is being expressed in the corresponding treatment group in average (row), and is color coded ranging from white (zero) to dark purple (one).

### VISR.

VISR was assessed 2 weeks after the third and fourth vaccinations and at 9 and 12 months after enrollment using a diagnostic algorithm that includes 4 different enzyme immunoassays (EIAs): Abbott Architect HIV Ag/Ab Combo, Abbot Prism, Bio-Rad Genetic Systems HIV Combo Ag/Ab EIA, and Bio-Rad Multispot HIV-1/HIV-2 Rapid Test. For participants with a positive result in any of these assays, RNA PCR (Abbott m2000 HIV-1 Real-Time PCR) was performed to distinguish vaccine-induced responses from actual infection.

### Statistics.

The sample size of 26 vaccine recipients per group provided a 90% chance of observing at least 1 SAE if the true rate of such an event was 8.5% or higher; there was a 90% chance that we would not observe any SAE if the true rate was less than 0.4%. For immune responses, the precision with which the true response rate can be estimated from the observed data depended on the true underlying response rate and the sample size. For example, the 2-sided 95% CI for the true response was approximately 67%–94% if the observed response rate was 85% based on the sample size of 26 vaccine recipients per group. The study had a limited power to detect response-rate differences between vaccine groups. The study could only detect a 30% or higher difference between vaccine groups with 80% power.

The percentages of participants experiencing each type of local and systemic reactogenicity sign or symptom are displayed using bar charts by severity and treatment group. For a given sign or symptom, each participant’s reactogenicity events were counted once under the maximum severity. Kruskal-Wallis tests were used to test for differences in severity across all treatment groups.

The distributions of the immune-response magnitudes are displayed by treatment group and visit, with responders in red dots and nonresponders in blue triangles and box-and-whisker plots based on the data from responders superimposed on the distribution. The midline of the box-and-whisker plot denotes the median and the ends of the box-and-whisker plot denote the 25th and 75th percentiles. The whiskers that extended from the top and bottom of the box extend to the most extreme data points that were no more than 1.5 times the interquartile range (i.e., height of the box) or if no value meets this criterion, to the data extremes. The positive immune response rates are displayed in bar charts by treatment group and time point in separate panels.

Positive immune response rates were compared between treatment groups using Fisher’s exact test and between visits within treatment groups using McNemar’s test. The immune-response magnitudes among positive responders were compared between treatment groups using Wilcoxon’s rank-sum exact test and between time points within treatment groups using Wilcoxon’s signed-rank test.

The trajectory of IgG/IgA and IgG subclass response rates over time are displayed by treatment group. The error bars at each time point are the 95% CI of the response rate. The 95% CI of the response rate was calculated using the score test method (42). The trajectory of IgG/IgA and IgG subclass response magnitudes over time are displayed by treatment groups in geometric means among all participants (responders and nonresponders) from each treatment group. The error bars at each time point are the 95% CIs of the geometric means by treatment group. The 95% CI of the geometric mean was calculated based on the normal distribution of log(IgG/IgA) and back-transformation.

MB plots characterize the magnitude (nAb titer) and breadth (number of isolates neutralized at a given titer) of each individual serum sample assayed against a panel of isolates. MB curves show, for each possible log_10_(ID_50_) titer threshold, the fraction of viruses with titers greater than this threshold. In addition to the individual sample-specific curves, the treatment-group-specific curve displays the average MB across all participants in that treatment group. The AUC-MB was calculated as the average of the log_10_(nAb) titer over the panel of isolates, where titers that are below the limit of detection were set to half of that limit. The AUC-MB was compared between treatment groups using Wilcoxon’s rank-sum exact test.

All *P* values are 2 sided and unadjusted for multiple comparisons. Given the nature and sample size of this phase II trial (*n* = 26 per treatment group), a somewhat increased type I (false positive) error without multiplicity correction was tolerated for better sensitivity (power) to detect the differences between treatment groups. Significant findings would generate the hypotheses for further testing in a larger trial in the future.

### Study approval.

The study was approved by the local institutional review boards (IRBs) at all participating sites: Human Research Protections Program/IRB, Vanderbilt University Medical Center Nashville, Tennessee, USA; University of Rochester Research Subject Review Board, Rochester, New York, USA; Fred Hutchinson Cancer Research Center IRB, Seattle, Washington, USA; UCSF Human Research Protection Program, San Francisco, California, USA; New York Blood Center, New York Blood Center IRB, New York, New York, USA; College of Physicians & Surgeons, Columbia University IRB, New York, New York, USA; University of Pennsylvania IRB, Philadelphia, Pennsylvania, USA. Clinical research site staff completed enrollment procedures and obtained written informed consent from all participants.

### Data availability.

A copy of the study protocol and the data underlying the findings of this manuscript can be found online at https://atlas.scharp.org/cpas/project/HVTN%20Public%20Data/HVTN%20105/begin.view?

## Author contributions

MCK, CM, NGR, RJ, SK, and ES designed the study. MES, IF, GJW, HVT, JM, AN, and MCK were responsible for the conduct of the trial. GDT, DCM, GF, SCDR, and MJM generated immunology data. SSL and BS conducted data analysis. FS, GP, and SD provided the vaccines used in the trial. NGR, MCK, and JK wrote the manuscript.

## Supplementary Material

Supplemental data

ICMJE disclosure forms

## Figures and Tables

**Table 1:**
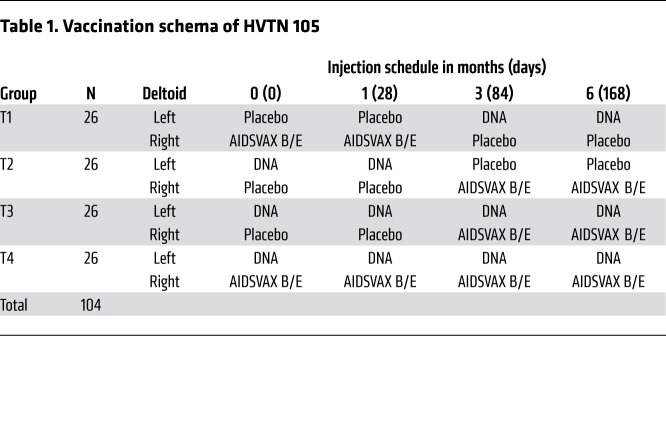
Vaccination schema of HVTN 105

## Version 1 09/30/2019

Electronic publication

## Version 2 11/01/2019

Print issue publication
