# Supplementary material for: DNA priming and gp120 boosting induces HIV-specific antibodies in a randomized clinical trial
Source: J Clin Invest. 2019 Sep 30;129(11):4769–85. doi: 10.1172/JCI128699 (PMC6819112; doi:10.1172/JCI128699)
Supplement: Supplemental data [file jci-129-128699-s186.pdf]

Supplemental Materials

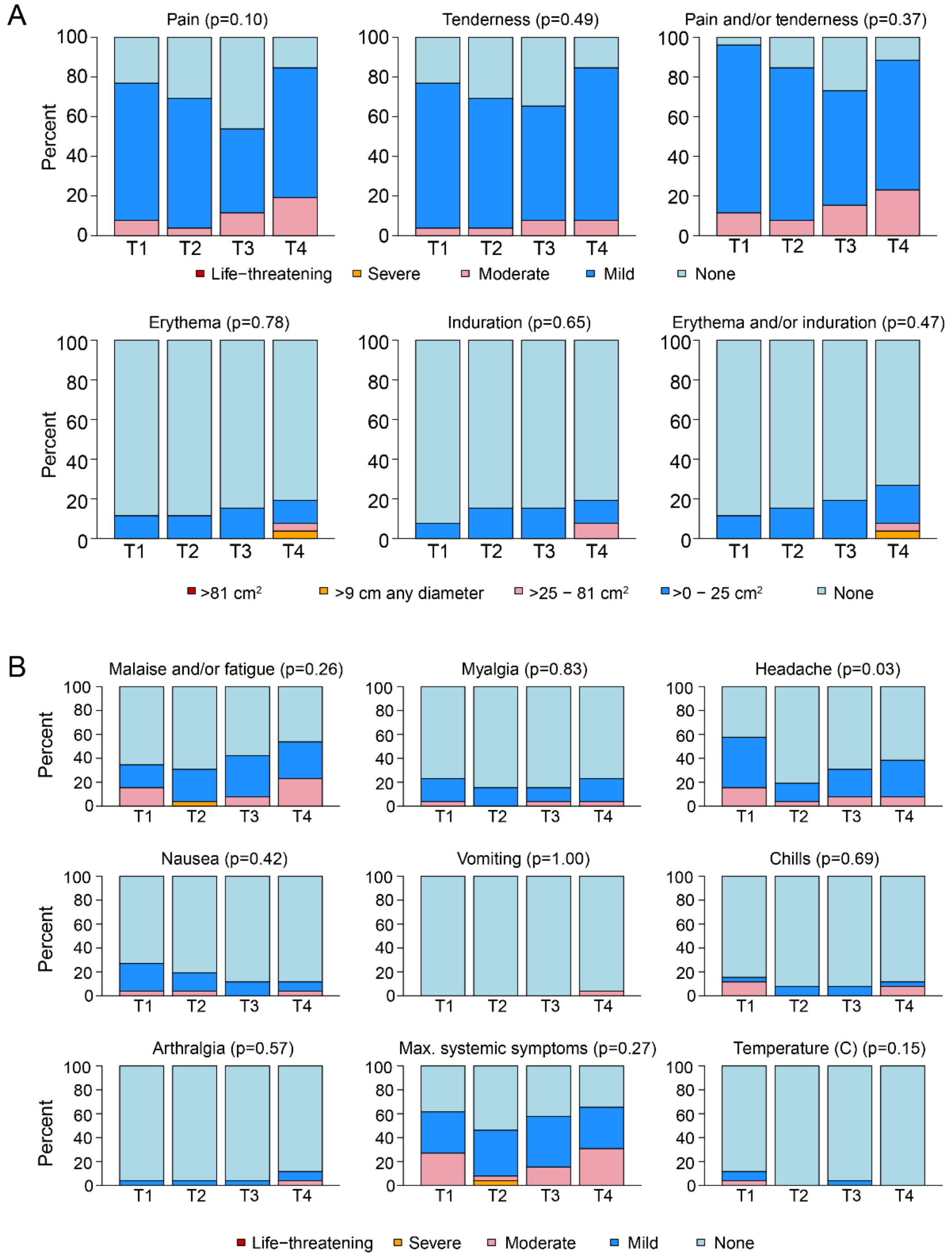

Supplemental Figure 1. Reactogenicity in HVTN 105. (A) Maximum local reactogenicity by treatment group in HVTN 105 (either deltoid). None were significant across the treatment groups (i.e., p-value ≥ 0.1). **(B)** Maximum systemic reactogenicity by treatment group in HVTN 105. Only

headache was significantly different across treatement groups (p=0.03). Kruskal-Wallis tests were used to compare the differences across the treatment groups (n=26 per treatment group).

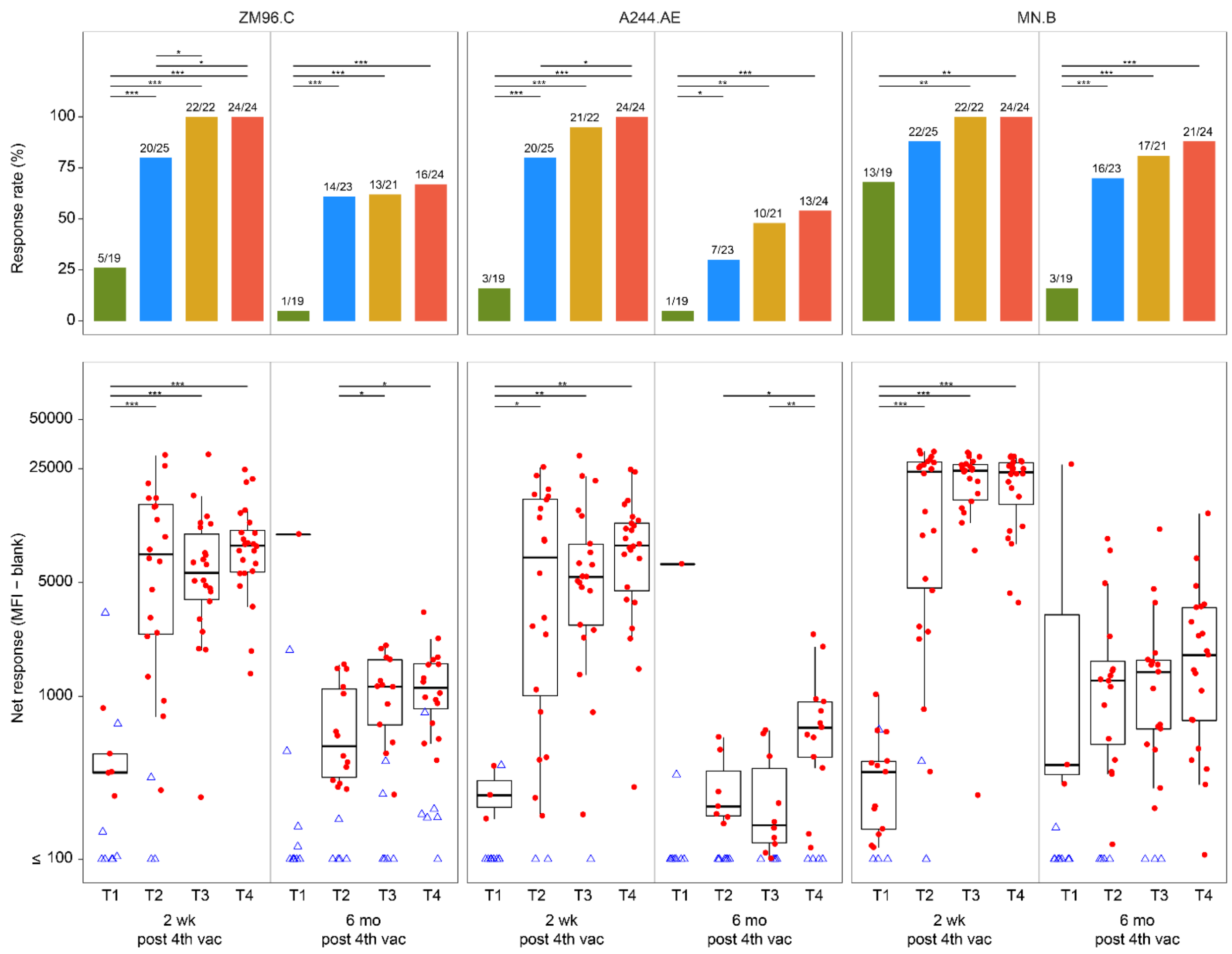

Supplemental Figure 2. IgG binding antibody responses against individual vaccine-matched antigens, as measured by binding antibody multiplex assay (BAMA) at two weeks and six months after the fourth vaccination in HVTN 105. Shown are the positive response rates (top panels) and the distribution of the response magnitudes (positive responders in red filled circles and non-responders in blue open triangles) and the boxplot among the positive responders (the mid-line of the boxplot denotes the median and the ends of the boxplot denote the 25th and 75th percentiles) (bottom panels) by timepoint and treatment group (n=25, 26, 26, 25 in T1-T4, respectively). Vaccine-matched antigens: ZM96.C, A444.AE, and MN.B. Bars and *s on top of boxplots indicate the significant comparisons between treatment groups (*: $P \leq 0.05$; **: $P \leq 0.01$; ***: $P \leq 0.001$) without multiple comparisons adjustments. The comparisons between treatment

groups were done using Fisher Exact test for response rates and Wilcoxon Rank Sum test for magnitudes. Fractions above bars indicate numbers of positive responders over total numbers of participants by timepoint and treatment group.

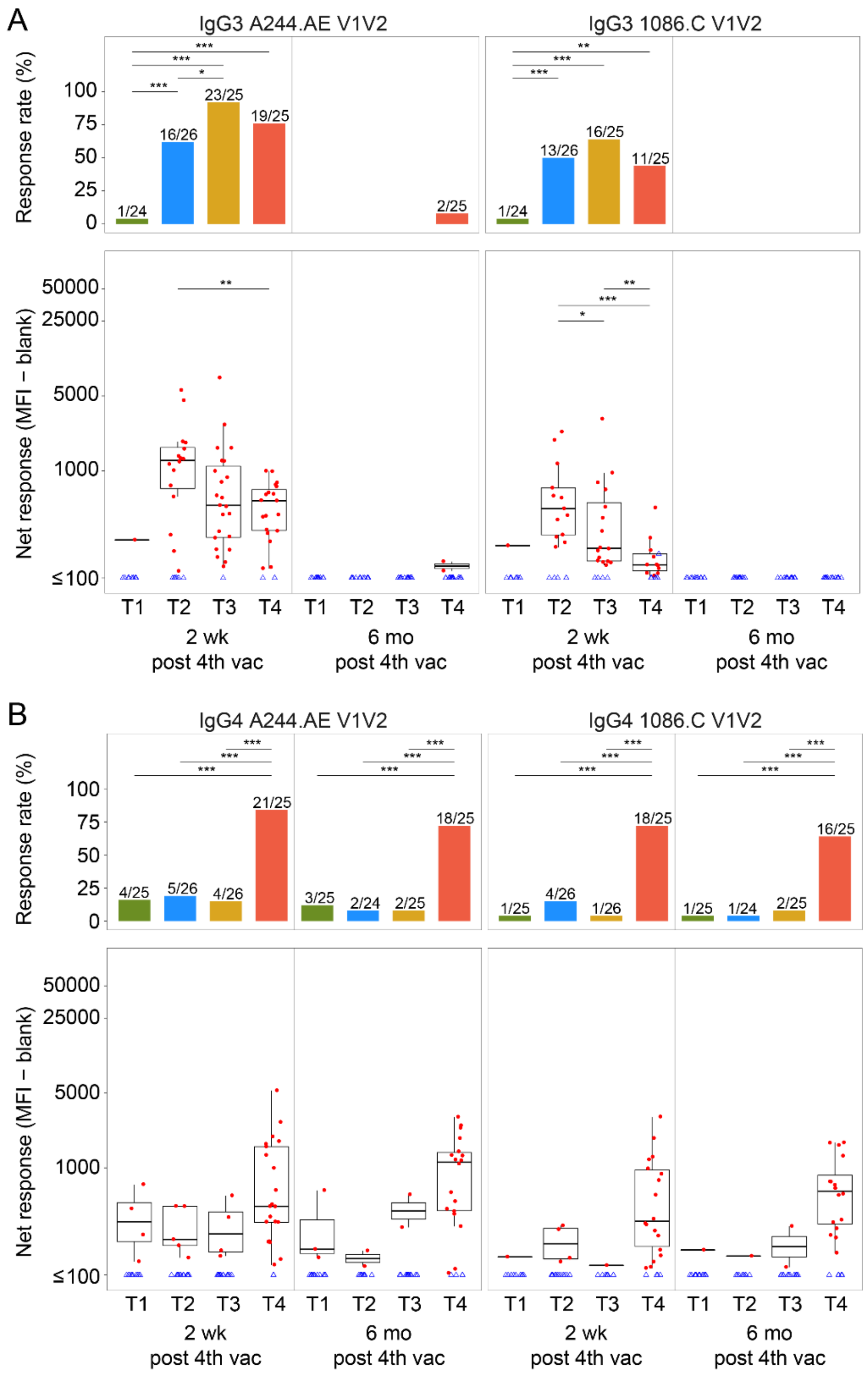

Supplemental Figure 3. IgG3 (A) and IgG4 (B) binding antibody responses against two V1V2 antigens, as measured by binding antibody multiplex assay (BAMA) at two weeks and six months after the fourth vaccination in HVTN 105. Shown are the positive response rates (top panelspanel) and the distribution of response magnitudes (positive responders in filled red circles

and negative responders in open blue triangles) and the boxplot (the mid-line of the boxplot denotes the median and the ends of the boxplot denote the 25th and 75th percentiles) among positive participants (bottom panels) by timepoint and treatment group (n=25, 26, 26, 25 in T1-T4, respectively). Subtype AE V1V2: A244.AE V1V2; subtype C V1V2: 1086.C V1V2. Bars and *s on top of boxplots indicate the significant comparisons between treatment groups (*: $P \leq 0.05$; **: $P \leq 0.01$; ***: $P \leq 0.001$) without multiple comparisons adjustments. The comparisons between treatment groups were done using Fisher Exact test for response rates and Wilcoxon Rank Sum test for magnitudes. Fractions above bars indicate numbers of positive responders over total numbers of participants by timepoint and treatment group.

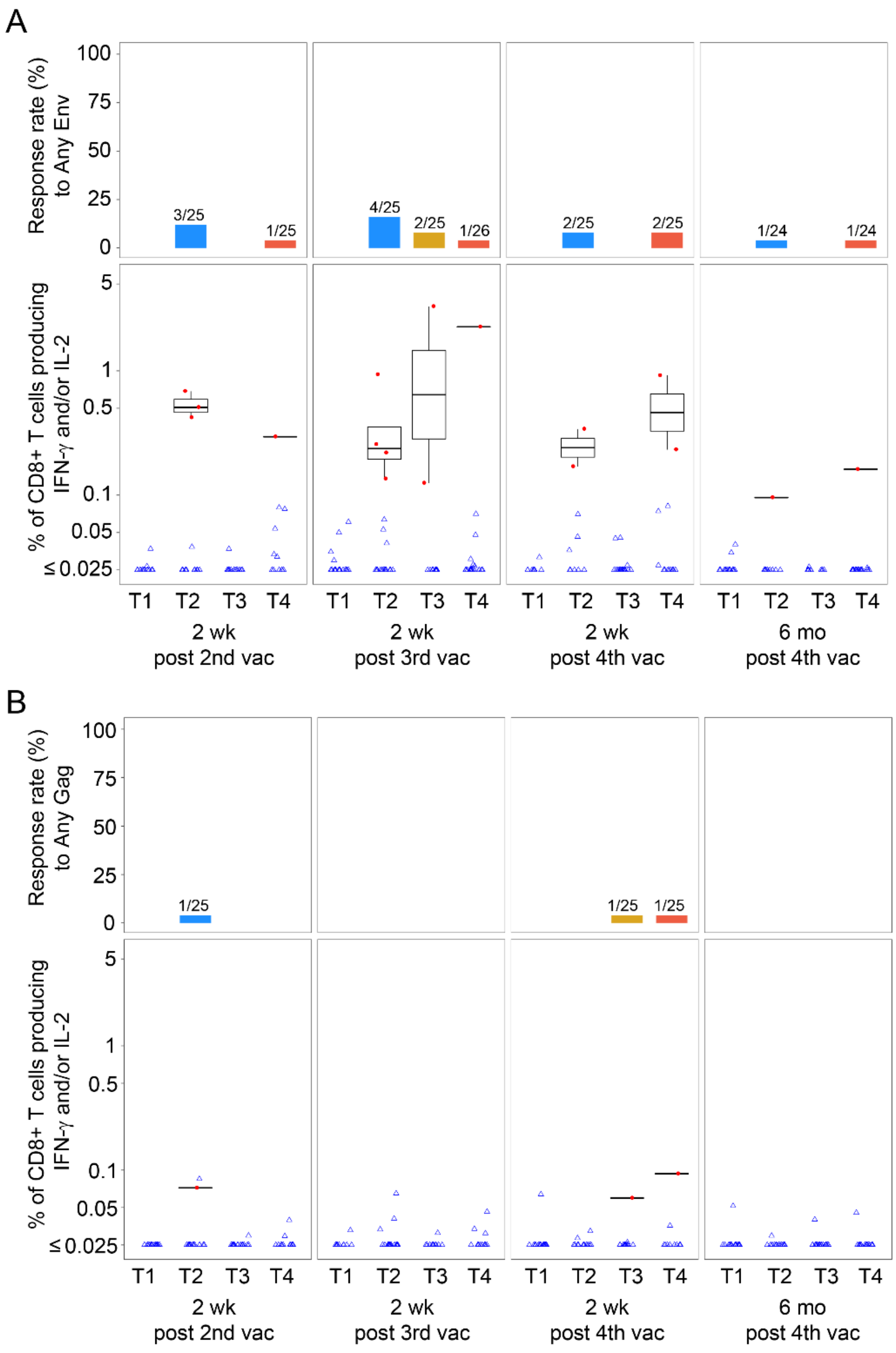

Supplemental Figure 4. CD8+ T-cell responses elicited in HVTN 105 measured by ICS and reported as the percentage of cells producing IFN-γ and/or IL-2 in each treatment group. **(A)** CD8+ T-cell responses to any HIV Env peptide pools all vaccine matched: ZM96 gp140 Env1, ZM96 gp140-Env2, and 92TH023-Env. **(B)** CD8+ T-cell responses to HIV Gag peptide pool: ZM96 Gag. Bar plots (on the top panels) show positive response rates by timepoint and treatment

group (n=25, 26, 25, 25 in T1-T4, respectively). The bottom panels show the distribution of response magnitudes (positivep respondersin filled red circles, negative responses in open blue triangles) and the boxplot (the mid-line of the boxplot denotes the median and the ends of the boxplot denote the 25th and 75th percentiles) for the positive responders only. Fractions above bars indicate numbers of positive responders over total numbers of participants by timepoint and treatment group.

Supplemental Table 1. Demographics in HVTN 105

	**T1** (n = 26)	**T2** (n = 26)	**T3** (n = 26)	**T4** (n = 26)	**Total** (n = 104)
**Sex**					
Male	12 (46%)	13 (50%)	12 (46%)	18 (69%)	55 (53%)
Female	14 (54%)	13(50%)	14 (54%)	8 (31%)	49 (47%)
**Ethnicity**					
Hispanic or Latino	0 (0%)	3 (12%)	1 (4%)	6 (23%)	10 (10%)
Non-Hispanic or Latino	26 (100%)	23 (88%)	25 (96%)	20 (77%)	90 (90%)
**Race**					
White	14 (54%)	20 (77%)	21 (81%)	17 (65%)	72 (69%)
Black/African American	7 (27%)	2 (8%)	2 (8%)	3 (12%)	14 (13%)
Asian	1 (4%)	3 (12%)	0 (0%)	1 (4%)	5 (5%)
Native Hawaiian/ Pacific Islander	0 (0%)	0 (0%)	0 (0%)	0 (0%)	0 (0%)
Native American/Native Alaskan	0 (0%)	0 (0%)	0 (0%)	1 (4%)	1 (1%)
Multiracial	3 (12%)	1 (4%)	3 (12%)	2 (8%)	9 (9%)
Other	1 (4%)	0 (0%)	0 (0%)	2 (8%)	3 (3%)
**Age**					
Median	28.0	26.0	27.0	26.0	27.0
Range	19-50	18-50	19-48	19-46	18-50

Supplemental Table 2. End of Study Diagnostic Testing Results in HVTN 105

GROUPGROUP	ASSAY TYPE	KIT NAME	REACTIVITY RATE	95% CONFDENCE INTERVAL
**T1**	Any	Vaccine-induced seroreactivity	1/26 = 3.8%	(0.7%, 18.9%)
	ELISA	Abbott Architect HIV Ag/Ab Combo	0/26 = 0.0%	(0.0%, 12.9%)
	ELISA	Abbott Prism	1/26 = 3.8%	(0.7%, 18.9%)
	ELISA	BioRad GS HIV Combo Ag/Ab EIA	0/26 = 0.0%	(0.0%, 12.9%)
	ELISA	BioRad Multispot HIV-1/HIV-2 Rapid Test	0/26 = 0.0%	(0.0%, 12.9%)
**T2**	Any	Vaccine-induced seroreactivity	1/26 = 3.8%	(0.7%, 18.9%)
	ELISA	Abbott Architect HIV Ag/Ab Combo	0/26 = 0.0%	(0.0%, 12.9%)
	ELISA	Abbott Prism	0/25 = 0.0%	(0.0%, 13.3%)
	ELISA	BioRad GS HIV Combo Ag/Ab EIA	0/26 = 0.0%	(0.0%, 12.9%)
	ELISA	BioRad Multispot HIV-1/HIV-2 Rapid Test	1/26 = 3.8%	(0.7%, 18.9%)
**T3**	Any	Vaccine-induced seroreactivity	1/26 = 3.8%	(0.7%, 18.9%)
	ELISA	Abbott Architect HIV Ag/Ab Combo	0/26 = 0.0%	(0.0%, 12.9%)
	ELISA	Abbott Prism	1/26 = 3.8%	(0.7%, 18.9%)
	ELISA	BioRad GS HIV Combo Ag/Ab EIA	0/26 = 0.0%	(0.0%, 12.9%)
	ELISA	BioRad Multispot HIV-1/HIV-2 Rapid Test	0/26 = 0.0%	(0.0%, 12.9%)
**T4**	Any	Vaccine-induced seroreactivity	3/26 = 11.5%	(4.0%, 29.0%)
	ELISA	Abbott Architect HIV Ag/Ab Combo	0/26 = 0.0%	(0.0%, 12.9%)
	ELISA	Abbott Prism	2/25 = 8.0%	(2.2%, 25.0%)
	ELISA	BioRad GS HIV Combo Ag/Ab EIA	0/26 = 0.0%	(0.0%, 12.9%)
	ELISA	BioRad Multispot HIV-1/HIV-2 Rapid Test	1/26 = 3.8%	(0.7%, 18.9%)

Supplemental Table 3. Details of the BAMA, ICS, and nAb antigens, including HIV-1 viral strain information

Assay	Antigen class	Full antigen name	Antigen name used in plot and throughout text	Viral strain information: Subtype.Country.Year.Stage*
BAMA	gp140	Con S gp140 CFI	Con S gp140 CFI	[Group M Consensus]
		A1.con.env03 140 CF	Consensus A gp140	A1.xx.xx.6
	gp120	A244 gp120 gDneg/293F/mon	A244.AE	CRF01_AE.TH.90.6
		MN gp120 gDneg/293F	MN.B	B.US.87.6
		96ZM651.D11gp120.avi	ZM96.C	C.ZM.96.6
		Con 6 gp120/B	Con 6 gp120	[Group M Consensus]
	V1V2	C.1086_V1_V2 Tags	1086.C V1V2	C.MW.04.1-2
		gp70_B.CaseA_V1_V2	CaseA2_gp70_V1V2.B	B.US.88.6
		gp70-B.CaseA2 V1/V2/169K	CaseA2_V1/V2/169K.B	B.US.88.6
		AE.A244 V1V2 Tags/293F	A244.AE V1V2	CRF01_AE.TH.90.6
		gp70-96ZM651.02 V1v2	ZM96.C V1V2	C.ZM.96.6
ADCC		96ZM651_D11gp120.avi293F	ZM96.C	C.ZM.96.6
		A244_D11/gp120_avi	A244.AE	CRF01_AE.TH.90.6
		B.MN_gDneg-gp120/293F	MN.B	B.US.87.6
ICS		ZM96 gp140-Env 1, ZM96 gp140-Env 2, 92TH023-Env pool	Any Env	-
		ZM96 Gag pool	Any Gag	-
TZM-Bl nAb	EPV**	BaL.26	BaL.26.B	B.US.85.6
		MN.3	MN.3.B	B.US.84.6
		MW965.26	MW965.26.C	C.MW.93.6
		SF162.LS	SF162.LS.B	B.US.89.6
		TH023.6	TH023.6.AE	CRF01_AE.TH.92.6

*Subtype is denoted by a capital letter; country of origin is denoted by the 2 digit International Organization for Standardization code; year isolated is denoted by 2 digits; when country of origin and year isolated are unknown, they are denoted as “xx”; and stage is denoted by “a” (acute, if Fiebig stage is unknown) or “1”, “2”, “3”, “4”, “5”, or “6” (acute or early chronic, where the number or range corresponds to the Fiebig stage or range of stages when known).
**EPV = Env-pseudotyped virus

Supplemental Acknowledgements

For the HVTN 105 Protocol Team and the NIAID HIV Vaccine Trials Network we thank Michael Keefer, Catherine Bunce, Sheila Bello-Irizarry (University of Rochester, New York, United States of America); Nadine Rouphael (Emory University, Atlanta, Georgia, United States of America); Cecilia Morgan, Sue Li, Brittany Sanchez, Shelly Karuna, Jim Maynard, Jill Zeller, Carissa Karg, Eva Chung, On Ho, Ryan Jensen, Scharla Estep, Renee Rivers, Hongwei Wang, Gina Escamilla, Lisa Sunner, Adi Ferrara, Lawrence Corey, Peter Gilbert, M. Juliana McElrath (Fred Hutchinson Cancer Research Center, Seattle, Washington, United States of America); Angela Broad (San Francisco Department of Public Health, San Francisco, California, United States of America; Robert Bucklew (Case Western Reserve University, Cleveland, Ohio, United States of America); Edith Swann, Laissa Ouedraogo (Division of AIDS, NIH, Bethesda, Maryland, United States of America); Carter Lee, Faruk Sinangil, Vineeta Gulati (Global Solutions for Infectious Diseases, South San Francisco, California, United States of America), Giuseppe Pantaleo (Lausanne University Hospital (CHUV), Lausanne, Switzerland); Song Ding (EuroVacc Foundation, Lausanne, Switzerland); Charla Andrews (US Military HIV Research Program, Bethesda, Maryland, United States of America).
